# Cognitive outcomes of deep brain stimulation depend on age and hippocampal connectivity in Parkinson's and Alzheimer's disease

**DOI:** 10.1002/alz.70498

**Published:** 2025-08-21

**Authors:** Calvin W. Howard, Martin Reich, Lan Luo, Niels Pacheco‐Barrios, Ron Alterman, Ana Sofia Rios, Michelle Guo, Ziyue Luo, Helen Friedrich, Andrew Pines, Leila Montaser‐Kouhsari, William Drew, Lauren Hart, Garance Meyer, Nanditha Rajamani, Maximillian U. Friedrich, Vanessa Milanese, Andres Lozano, for the ADvance Study Research Group, Thomas Picht, Katharina Faust, Andreas Horn, Michael D. Fox

**Affiliations:** ^1^ Center for Brain Circuit Therapeutics Brigham & Women's Hospital, Harvard Medical School Boston Massachusetts USA; ^2^ Department of Neurology Brigham & Women's Hospital, Harvard Medical School Boston Massachusetts USA; ^3^ Klinik für Neurologie mit Experimenteller Neurologie, Charité – Universitätsmedizin Berlin Berlin Germany; ^4^ Clinician Investigator Program, Postgraduate Medical Education University of Manitoba Winnipeg Manitoba Canada; ^5^ Department of Neurology University Clinic of Würzburg Würzburg Germany; ^6^ Department of Neurology Beth Israel Deaconess Medical Center, Harvard Medical School Boston Massachusetts USA; ^7^ Department of Neurosurgery Brigham and Women's Hospital, Harvard Medical School Boston Massachusetts USA; ^8^ Carrera de Medicina Humana Universidad Científica del Sur Lima Peru; ^9^ Department of Neurosurgery Beth Israel Deaconess Medical Center, Harvard Medical School Boston Massachusetts USA; ^10^ Department of Psychiatry Brigham & Women's Hospital, Harvard Medical School Boston Massachusetts USA; ^11^ Center of Neurology and Neurosurgery Associates (NeuroCENNA) BP – A Beneficência de São Paulo São Paulo Brazil; ^12^ Department of Surgery (Neurosurgery) University of Toronto Toronto Ontario Canada; ^13^ Department of Neurosurgery Charité – Universitätsmedizin Berlin Berlin Germany; ^14^ Department of Neurosurgery Massachusetts General Hospital, Harvard Medical School Boston Massachusetts USA; ^15^ Movement Disorders and Neuromodulation Unit, Department of Neurology Charité – Universitätsmedizin Berlin Berlin Germany; ^16^ Einstein Center for Neurosciences Berlin Charité – Universitätsmedizin Berlin Berlin Germany; ^17^ Athinoula A. Martinos Center for Biomedical Imaging Department of Radiology Massachusetts General Hospital, Harvard Medical School Charlestown Massachusetts USA

**Keywords:** Alzheimer's disease, cognition, connectomic deep brain stimulation, deep brain stimulation, Parkinson's disease

## Abstract

**INTRODUCTION:**

Here we contrast cognitive outcomes of deep brain stimulation (DBS) in Parkinson's disease (PD) with Alzheimer's disease (AD) to isolate the shared effect of DBS upon cognition while filtering out disease‐specific effects. Based on prior literature, we evaluate how DBS connectivity to the hippocampus influences cognition. We then evaluate how patient factors moderate this relationship.

**METHODS:**

We studied electrode locations and cognitive outcomes in patients who received subthalamic nucleus (STN) DBS for PD (2 datasets: *n* = 33, *n* = 28) or fornix DBS for AD (1 dataset: *n* = 46). We then investigate the moderating effect of patient factors and similarities across diseases.

**RESULTS:**

DBS site connectivity to the hippocampus was cognitively deleterious in PD but beneficial in AD. The opposite findings were driven by patient age. This effect was mediated by age‐related hippocampal atrophy.

**DISCUSSION:**

The shared cognitive effects of DBS across PD and AD depend on hippocampal connectivity and age.

**Highlights:**

- Cognition can be positively or negatively modulated in the same manner across diseases.
- Contrary to current clinical practice, older Parkinson's disease (PD) patients may benefit from deep brain stimulation (DBS).
- Our results support limiting enrollment to patients over 65 for Alzheimer's disease (AD) DBS, an emerging therapy currently in a phase 3 clinical trial.
- Hippocampal volume mediates the impact of DBS, suggesting patient atrophy must be considered in patient‐specific care.

## 1 BACKGROUND

Deep brain stimulation (DBS) to the subthalamic nucleus (STN) is effective for treating most motor symptoms of Parkinson's disease (PD). However, STN DBS can also lead to side effects, including DBS‐induced cognitive decline.^1^, ^2^, ^3^, ^4^, ^5^ The reported incidence of this cognitive decline is highly variable in the published literature, ranging from 5% to 40% of patients.^6^ Additionally, cognitive decline post DBS is challenging to detect, the extent is difficult to characterize, it can impact multiple cognitive domains, and there is no agreement on the best choice of test to measure the decline.^7^ However, one potential risk factor is the DBS site's connectivity to brain networks involved in cognition. One such cognitive network, defined by connectivity to the subiculum,^8^ has been associated with multiple different aspects of cognition including DBS‐induced general cognitive impairment,^8^ reversible impairment in executive tasks,^8^ and memory impairment in strokes. Although there are likely many other factors contributing to DBS‐induced cognitive decline,^9^, ^10^, ^11^, ^12^, ^13^, ^14^, ^15^ connectivity to the subiculum is a cognitive decline risk factor that could be modified with DBS re‐programming.^8^, ^16^

DBS to the fornix is also an experimental treatment for patients with Alzheimer's disease (AD) and has been applied to one cohort worldwide.^17^, ^18^, ^19^ Recent work suggests that DBS sites associated with the greatest cognitive outcomes in AD are connected to a distributed brain circuit, including regions in the hippocampus.^20^

These results seem to be the opposite of the above finding in PD, creating an apparent paradox.^8^ How can DBS sites connected to the hippocampus cause cognitive decline in PD, but lead to better cognitive outcomes in AD? The answer is not in the DBS parameters, as both treatments use similar frequency, amplitude, and pulse width.^8^, ^17^ Potential explanations lay in patient factors which have previously been shown to influence DBS cognitive outcomes, including baseline cognition in PD, sex in PD, and age in PD and AD.^12^, ^21^, ^22^ Both the PD and AD literature suggest that age may play a role in post‐DBS cognitive outcomes; however, older age has been associated with worse cognitive outcome in PD but better cognitive outcomes in AD.^11^, ^23^

The goal of this study was to investigate an apparent paradox in the current literature: how can STN DBS sites connected to the hippocampus result in cognitive decline while fornix DBS sites connected to the hippocampus result in cognitive benefit. To try and resolve this paradox, we performed the first study of DBS effects on cognition combining patients with PD and AD, contrasting each disease to identify shared effects of DBS upon cognition while filtering out disease‐specific effects. We tested three specific hypotheses that might explain this apparent paradox: (1) the connections responsible for worse cognitive outcomes in PD but better cognitive outcomes in AD appear similar, but are actually different; (2) DBS effects depend on baseline cognition, which is worse in AD and better in PD; and (3) DBS effects depend on patient age, which is higher in AD and lower in PD.^21^, ^24^, ^25^ To test these hypotheses, we measured connectivity of the DBS sites in AD and PD patients to the subiculum, related subiculum connectivity to cognitive outcomes, and investigated which patient factors may influence this relationship.

## 2 METHODS

### 2.1 Subjects: PD discovery cohort

The PD discovery cohort included 33 subjects who underwent STN DBS in Wurzburg, Germany and have been described previously.^8^, ^26^ DBS parameters were 150 Hz, pulse width 50 µs, and 3.5 mA using Medtronic 3387 or 3389 electrodes. Participants in this cohort were aged between 46 and 79 years, with a mean age of 58 ± 8 years. Exclusion criteria were defined and applied in the original study, and involved lead trajectories passing through the head of the caudate, as this has been associated with cognitive decline.^27^ No further patients were excluded in the present study. Cognitive outcomes were Mattis Dementia Rating Scale (MDRS) at baseline and 1 year follow‐up. Cognition was measured pre‐operatively and then 1 year post‐operatively in the ON med / ON stim condition.

### 2.2 Subjects: AD discovery cohort

The AD cohort comprised 46 participants receiving fornix DBS, enrolled as part of the multi‐site ADvance study conducted in the United States and Canada.^17^ DBS parameters were 130 Hz, pulse width 90 µs, and 3.5 mA using Medtronic 3387 electrodes. Subjects ranged in age from 47 to 79 years, with a mean age of 68 ± 8 years. Inclusion criteria were defined and applied in the original study and mandated a diagnosis of probable AD dementia in accordance with National Institute on Aging/Alzheimer's Association guidelines.^28^ No further patients were excluded in the present study. Patients exhibited mild dementia, evidenced by a Clinical Dementia Rating Sum of Boxes score of 0.5 or 1, and Alzheimer's Disease Assessment Scale‐13 scores ranging from 12 to 24. All participants had a designated caregiver capable of reliably reporting on the patient's daily activities and functioning. Exclusion criteria included significant neuropsychiatric symptoms, indicated by a Neuropsychiatric Inventory total score >n 10 or > 4 in any domain, except for apathy. Cognitive outcomes were measured by Alzheimer's Disease Assessment Scale‐11 (ADAS‐Cog 11) at baseline and 1 year follow‐up. Cognition was measured pre‐operatively and then 1 year post‐operatively ON med / ON stim condition.

**RESEARCH‐IN‐CONTEXT**

1. **Systematic review**: We reviewed prior publications and reference lists on deep brain stimulation (DBS) to the subthalamic nucleus (STN) and fornix in Parkinson's disease (PD) and Alzheimer's disease (AD). These studies highlighted hippocampal connectivity as a key driver of DBS‐related cognitive changes.
2. **Interpretation**: Our multi‐cohort analysis reconciles why DBS sites connected to the hippocampus can worsen cognition in younger PD patients yet improve cognition in older AD patients. Age‐related hippocampal atrophy mediates these divergent outcomes, unifying DBS‐induced cognitive effects across diseases.
3. **Future directions**: Prospective trials are needed to confirm the roles of age, hippocampal volume, and DBS connectivity. Such studies may refine stimulation targets and programming strategies that personalize DBS therapy for both PD and AD, maximizing cognitive benefits and minimizing harm.

### 2.3 Subjects: PD validation cohort

To validate results observed in our discovery cohorts, an additional cohort of 28 STN DBS PD patients was recruited from Boston, Massachusetts, USA. DBS parameters were 130 Hz, pulse width 60 µs, and 3.5 mA using Medtronic 3387 electrodes. There were no inclusion/exclusion criteria associated with the original data, and no further patients were excluded in the present study. No patients have an electrode passing through the head of the caudate. The cohort consisted of patients aged between 48 and 78 years, with a mean age of 62 ± 7 years. These patients received the Movement Disorder Society Unified Parkinson's Disease Rating Scale Part I (UPDRS‐I), with cognitive outcomes measured by question 1—Cognitive Impairment. The scale consisted of patient subjective evaluations from 0 (no impairment) to 4 (severe impairment). Patients were assessed at baseline and 1 year later. Cognition was measured pre‐operatively and then 1 year post‐operatively ON med / ON stim condition.

### 2.4 Standardization of cognitive scores

Comparison of cognitive outcomes across PD and AD cohorts required comparison across different cognitive scales.^29^, ^30^ To compare cognitive outcome, the percent cognitive improvement from baseline to 1 ‐year follow‐up was calculated. These percent‐improvement scores were then standardized with *z* scoring. This converts all cognitive metrics to a similar distribution centered around their mean cognitive response.

Baseline cognition is the patient's cognition at their pre‐operative evaluation. To compare across diseases, values were normalized and set such that a lower baseline represented better clinical status. This process enabled direct comparison but did not alter the distributions.

### 2.5 DBS reconstruction

Patient electrodes were reconstructed using Lead‐DBS as previously described (Supplementary Materials 1.1, Figure S1 in supporting information).^8^, ^20^, ^31^ Briefly, post‐operative computed tomography and magnetic resonance imaging scans from each patient were registered to Montreal Neurologic Institute (MNI) space using Advanced Normalization Tools.^32^ In the AD population, atrophy‐induced registration errors were fixed manually using WarpDrive.^33^ Electrodes were reconstructed using the TRAC algorithm with subsequent manual refinement if necessary.^31^

Next, Lead‐DBS was used to model the stimulation volumes in each patient in accordance with previously published methods.^31^ Briefly, the electrical field being generated by the active contact of the DBS electrode was calculated from the patient's stimulation parameters.^34^ Then, the electrical field meeting sufficient magnitude to depolarize surrounding tissue was calculated.^35^ The resulting three‐dimensional region represents the volume of tissue activated (VTA) for each patient (Figure S1).

### 2.6 Definition of subiculum–retrosplenial cortex region of interest

We used an a priori region of interest in the subiculum–retrosplenial cortex, which was initially derived based on the connectivity of brain lesions causing memory impairment.^36^ This same region of interest was used in a subsequent publication on the connectivity of STN DBS sites causing cognitive decline.^8^, ^36^

### 2.7 Measurement of connectivity

A normative human connectome was used to derive connectivity between each patient's VTA and the subiculum as described previously (Supplementary Materials 1.1).^8^, ^37^ Briefly, we calculated seed‐based functional connectivity between each VTA and the subiculum region of interest using resting‐state functional connectivity data (2 × 2 × 2‐mm resolution) from 1000 healthy participants (human brain connectome from the Brain Genomics Superstruct Project).^38^, ^39^ This method calculates the average blood oxygen level–dependent signal within each patient's VTA, extracts the time course of changes in this signal, and performs a Pearson correlation with the extracted time course from the subiculum region of interest. Results are combined across the 1000 subjects, resulting in a single *t* value, which we refer to as “connectivity” between the VTA and the subiculum. For each patient, we considered the left and right VTAs as a single region of interest for connectivity analyses, effectively averaging connectivity of both VTAs.

### 2.8 Relating subiculum connectivity to cognitive outcomes

Connectivity between each patient's VTA and the subiculum was related to cognitive outcome using Pearson correlation. To ensure results were robust to outliers this analysis was repeated using Spearman correlation. Significant differences in either correlation value between two cohorts (e.g., PD vs. AD) were identified using permutation testing, as in prior work.^40^ Specifically, the absolute difference between two correlation values was computed, then re‐computed 10,000 times after randomly shuffling cognitive outcomes across different patients within a cohort (Figure S2 in supporting information). We term this a delta‐R analysis.

### 2.9 Data‐driven subiculum connections correlating to cognitive outcome

For each cohort, we performed a data‐driven analysis of the DBS connections most associated with cognitive outcomes, generating R‐maps as have been previously described.^20^ Briefly, R‐maps are generated by correlating the connectivity profiles of each DBS electrode with cognitive outcomes.

To evaluate if the subiculum connections covarying with cognitive outcomes were different across diseases, we generated R‐maps and compared the within‐subiculum peaks across both diseases. Specifically, we measured peak‐to‐peak distance. Then, we evaluated if this distance was smaller than expected by chance with permutation testing. Cognitive outcomes were shuffled, R‐maps were recalculated, and the distance between subiculum peaks was measured multiple times (10,000 permutations). The number of times the peak‐to‐peak distance matched or was smaller than our measured distance composed the *p* value.

To evaluate whole‐brain similarity, we measured their spatial correlation and applied permutation testing to see if they were more similar than expected by chance. Permutation testing was done by shuffling each patient's cognitive outcomes (within diseases), recalculating the R‐maps, and measuring their spatial correlation (10,000 permutations).

### 2.10 Relating connectivity, patient factors, and cognition

We used multiple regression to relate cognitive outcomes with regressor variables such as subiculum connectivity, age, and cognitive baseline. These regressions can be visualized as a response surface.^41^, ^42^, ^43^ The response surface is the estimated outcome across all possible regressor values.^41^ This approach extends the regression line for two variables (2D) to a plane for multiple variables (3D+).

The response surface allows intuitive visualization of how the regressors influence the overall regression. When regressors do not interact, the surface is flat. When the regressors do interact, the surface becomes curved.^42^

We were interested in if PD and AD had a similar relationship with cognitive outcomes and regressor variables. This was assessed in two ways. First, a parametric goodness‐of‐fit test can be performed, which assessed if the regression from one dataset accurately fit the second dataset. Second, a non‐parametric method compares the similarity of the response surface of the regression between PD and AD. The similarity is the spatial correlation of the two response surfaces. To evaluate if the similarity of the surfaces is greater than expected by chance, we use permutation testing. The permutations shuffle cognitive outcomes within each disease, recompute the response surfaces, and measure their similarity (*n* = 10,000).

### 2.11 Deriving age and connectivity subgroups

We split patients into age‐based subgroups using a cutoff of 65. This age cutoff was chosen based on the existing AD DBS literature,^25^ in which an age‐based cutoff has been defined and used in a phase 3 clinical trial.^25^ While there is no clear age‐based cutoff for cognitive outcomes in the PD DBS literature, one study has suggested age 70.^44^ As such, we used age 65 as our primary cutoff, confirmed results were unchanged if we used age 70 as a cutoff, then derived data‐driven age inflection points to ensure that all results were independent of these cutoffs.^44^, ^45^

We also derived the inflection points in a data‐driven manner. We solved the regressions relating age, subiculum connectivity, and cognitive outcomes for the inflection point in age and connectivity (Equation S1 in supporting information). These data‐driven inflection points can then be used to define subgroups above or below the inflection. Solving for the inflection point requires solving for the partial derivative of cognitive outcomes with respect to age or connectivity (Equation S2 in supporting information) and solving for the inflection point (Equation S3 in supporting information). The solution yields the inflection point in age (in years), and the inflection in connectivity (in *t* values), which represent the value at which age or connectivity transition from being beneficial to impairing while holding the other variable constant. Additionally, to assess for robustness, we solved for the inflection points in these variables with the empiric gradient of the response topology (Supplementary Materials 1.2 in supporting information).

### 2.12 Comparison across inflection groups

Patients were split into age groups using the literature‐based age inflection points (65, 70), and delta‐R analyses were performed to evaluate differences in how these groups responded to subiculum connectivity. This was then repeated with the data‐driven inflection points in age (discovery PD = 64.7 years, discovery AD = 65.3 years), in which AD was used to binarize PD and vice versa.

This process was then repeated, but patients were split into connectivity groups using the data‐driven inflection point in subiculum (discovery PD = 24.2 T, discovery AD = 23.8 T). Inflection points derived from the discovery cohorts were applied to the validation cohort.

### 2.13 Characterizing subiculum connectivity within the STN and fornix

A normative human connectome was used to derive connectivity between the subiculum and all voxels within the STN and fornix as described above. This connectivity analysis estimated subiculum connectivity across all STN and fornix voxels for 1000 subjects. These subiculum connectivity values were then averaged, generating the mean estimate of subiculum connectivity within the STN and fornix. The data‐driven connectivity inflection was then used to subgroup STN and fornix voxels into “low” and “high” subiculum connectivity. We then project these regions onto anatomic atlases of the STN and fornix in template space for a schematic visualization.

### 2.14 Quantification of hippocampal volume

Patient hippocampal gray matter volumes were processed using the Computational Anatomy Toolbox as has been previously described.^46^ Briefly, patient T1 scans were segmented into cerebrospinal fluid, white matter, and gray matter. Gray matter volume within the hippocampus was estimated using each patient's hippocampal parcellation via the Neuromorphometrics atlas.^46^, ^47^ Hippocampal gray matter was corrected for total intracranial volume.

### 2.15 Statistics statement

Statistical analyses, including mediation analyses, were conducted in Python 3.10 using Statsmodels 0.14.0, Nilearn 0.10.1, and Scikit‐learn 1.3.0.^48^, ^49^, ^50^ Descriptive statistics are presented as mean ± standard deviation. Correlations used Spearman and Pearson methods as appropriate. Ordinary least squares generalized linear models (GLMs) were used for assessing interaction term significance, validated by *F* tests comparing reduced versus interacting models. All models were fit on standardized and unstandardized data, but standardized *p* values are reported given improved statistical validity. Bonferroni correction was used to correct *p* values for multiple comparisons. All permutation tests were done with 10,000 permutations. Cohorts were controlled for in all analyses. In GLMs, cohort was added as a covariate. In delta‐R analyses, each cohort was assessed individually. Total delta‐R analyses summed the difference across groups. Additionally, mixed effects analyses were used to account for the random slopes and intercepts of each cohort.

Bootstrapping was used to estimate the true distribution of correlation values. Briefly, bootstrapping is a technique in which the patient sample is resampled, allowing patients to occur multiple times in a resample. This allows multiple variants of a potential sample to be created, and the statistic of interest (correlation coefficient) is recomputed for each sample. Statistically, this is used to approximate the error in the true statistic of interest (correlation coefficient), and estimate what its distribution in the true population may be.^51^

## 3 RESULTS

**FIGURE 1:**
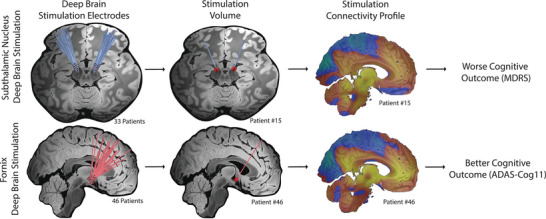
Deep brain stimulation in PD and AD can result in opposite cognitive outcomes. Top panel, 33 PD patients receiving subthalamic nucleus deep brain stimulation described by Reich in 2022.^8^ Bottom panel, 46 AD patients receiving fornix deep brain stimulation described by Ríos in 2022.^20^ The deep brain stimulation electrodes from these patients were reconstructed and the volume of stimulated tissue was estimated. Connectivity between each patient's stimulation site and other brain regions was computed. For PD patients, this connectivity profile results in worse cognitive outcomes, while for AD it results in better cognitive outcomes. AD, Alzheimer's disease; ADAS‐Cog11, Alzheimer's Disease Assessment Scale, 11‐item cognitive subscale; MDRS, Mattis Dementia Rating Scale; PD, Parkinson's disease.

The locations of DBS electrodes in each cohort were mapped to a common brain atlas and stimulation sites were computed for patients with PD (PD discovery cohort, *n* = 33, age 58 ± 8 years) who received DBS to the STN and patients with AD (AD discovery cohort, *n* = 46, age 68 ± 8) who received DBS to the fornix (Figure 1). A table of demographic factors for the cohorts is available in Table 1. These cohorts were selected because they have been used in prior papers relating DBS connectivity to worse cognitive outcomes in PD^8^ and better cognitive outcomes in AD (Figure S1).^20^

**TABLE 1:** Demographics and cohort factors. Cognitive scores in the PD discovery cohort are measured by the MDRS and range from 0 to 144. Cognitive scores in the AD discovery cohort are measured by the ADAS‐Cog scale and range from 0 to 70. Cognitive scores in the PD validation cohort are measured by the UPDRS‐1 scale and range from 0 to 5.

	PD discovery	AD discovery	PD validation
*n*	33	46	29
Baseline cognitive score, mean (SD)	141.3 (2.4)	19.2 (5.7)	1.2 (0.9)
1 Year follow‐Up cognitive score, mean (SD)	138.9 (4.5)	25.6 (9.9)	1.3 (1.0)
Cognitive outcome, *n* (%)			
Declined	28 (84.8)	37 (80.4)	22 (76)
Improved	5 (15.2)	9 (19.6)	7 (24)
Sex, *n* (%)			
Female	11 (33.3)	23 (50.0)	13 (44.8)
Male	22 (66.7)	23 (50.0)	16 (55.2)
Age, mean (SD)	58.2 (8.2)	68.0 (8.0)	62.0 (7.4)
Hippocampus GM volume (mL), mean (SD)	7.6 (1.7)	4.9 (0.9)	7.2 (1.9)
Subiculum connectivity (*t*), mean (SD)	22.6 (7.0)	25.0 (3.0)	23.2 (5.5)

Abbreviations: AD, Alzheimer's disease; ADAS‐Cog, Alzheimer's Disease Assessment Scale, cognitive subscale; GM, gray matter; MDRS, Mattis Dementia Rating Scale; PD, Parkinson's disease; SD, standard deviation; UPDRS‐1, Unified Parkinson's Disease Rating Scale Part 1.

### 3.1 DBS site connectivity to the same brain regions has opposite effects on cognition in PD and AD

**FIGURE 2:**
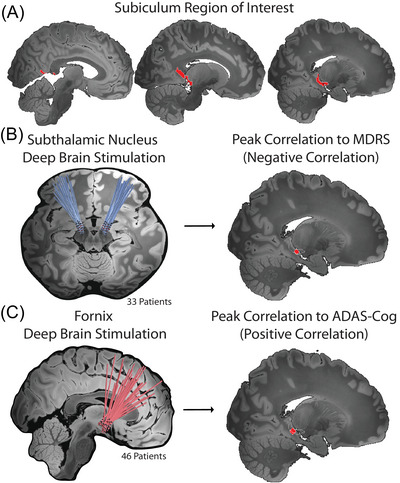
DBS site connections most strongly correlated with cognition fall within the same subiculum subregion in PD and AD. A, The a priori region of interest (ROI) used in this study, which spans the retrosplenial cortex to the subiculum of the hippocampus. B, Connectivity between STN DBS sites in PD (left, electrode locations shown in blue) and each voxel within the subiculum ROI was computed, and the peak correlation with cognitive outcome identified (right, red voxels). C, Connectivity between fornix DBS sites in AD (left, electrode locations shown in red) and each voxel within the subiculum ROI was computed, and the peak correlation with cognitive outcome identified (right, red voxels). In both (B) and (C), the subiculum ROI is shown for reference using a white outline. AD, Alzheimer's disease; ADAS‐Cog, Alzheimer's Disease Assessment Scale, cognitive subscale; DBS, deep brain stimulation; MDRS, Mattis Dementia Rating Scale; PD, Parkinson's disease; STN, subthalamic nucleus.

First, given reports of differences in hippocampal subnetworks, we investigated if seemingly opposite responses to subiculum connectivity could be driven by different subiculum connections.^52^ We performed a data‐driven search for connections covarying with cognition in PD and (separately) in AD (Figure 2). In both PD and AD, the peak connection covarying with cognition were just one voxel apart (2 mm, *p* = 0.0070), and fell inside Brodmann area 27,^36^ the subiculum (Table S1 in supporting information). Connectivity of one disease's DBS sites to the subiculum peak identified defined by the other disease was correlated with cognitive outcomes (but sign‐flipped, *p*_max_ = 0.0216). Whole‐brain connections covarying with cognition were also topologically similar, but opposite across PD and AD cohorts (*p* = 0.0072). Additional control analysis details are available (Supplementary Materials 1.3 in supporting information).

**FIGURE 3:**
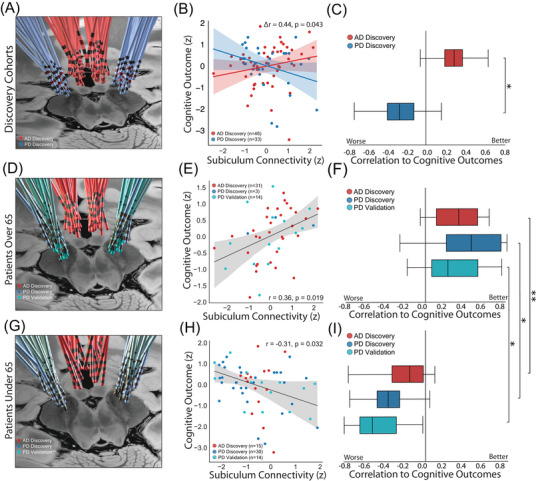
The paradoxical cognitive response between PD and AD patients to subiculum connectivity is driven by patient age. A, Electrode localizations for the PD and AD discovery cohorts, which demonstrated the original paradox. Active contacts are shown in red. B, Scatterplot relating subiculum connectivity and cognitive outcomes for PD and AD, with significantly different correlations by permutation test (ΔR = 0.51, *p* = 0.021). C, Boxplots demonstrating median and interquartile range of the bootstrapped correlations between subiculum connectivity to cognitive outcomes for AD and PD. D, Electrode localizations for older patients (age > 65) in the PD discovery, validation, and AD discovery cohorts. E, In older patients (age > 65) across the three DBS cohorts, subiculum connectivity was positively associated with cognitive outcomes (*r* = 0.36, *p* = 0.019). F, Boxplots demonstrating the bootstrapped correlations in older patients (age > 65) between subiculum connectivity and cognitive outcomes within each of the cohorts. G, Electrode localizations for younger patients (age < 65) in the PD discovery, validation, and AD discovery cohorts. H, In younger patients (age < 65) across the three DBS cohorts, subiculum connectivity was negatively associated with cognitive outcomes (*r* = –0.31, *p* = 0.032. I, Boxplots demonstrating the bootstrapped correlations in younger patients (age < 65) between subiculum connectivity and cognitive outcomes within each of the cohorts. Right, This difference in correlation between older and younger cohorts was significant across all three DBS cohorts (*R* = 0.67, *p* = 0.0091) as well as in each of the three DBS cohorts tested independently: AD discovery cohort (*R* = 0.55, *p* = 0.0073), PD discovery cohort (*R* = 0.80, *p* = 0.037), and PD validation cohort (*R* = 0.65, *p* = 0.029). All boxplots show mean and interquartile range of correlation coefficients from 10,000 bootstraps, with significance derived using permutation test (*: *p* < 0.05; **: *p* < 0.01). AD, Alzheimer's disease; ADAS‐Cog11, Alzheimer's Disease Assessment Scale, 11‐item cognitive subscale; DBS, deep brain stimulation; MDRS, Mattis Dementia Rating Scale; PD, Parkinson's disease.

We next investigated if the effect of connectivity to the entire subiculum region of interest truly differed across PD and AD cohorts, as this specific region was not tested in the original reports.^8^, ^20^ Connectivity of each patient's stimulation site to the entire a priori region of interest was computed and subsequently correlated with cognitive outcomes (Figure 3A–C). Connectivity to the same region in the subiculum was associated with worse cognitive outcomes in PD but better cognitive outcomes in AD, a difference which was statistically significant (ΔR = 0.51, *p* = 0.022). This result was consistent using either Pearson or Spearman correlation, using either the subiculum region of interest or a whole‐brain memory network map,^36^ and using standardized or non‐standardized cognitive scores (Supplementary Materials 1.4, Figure S3 in supporting information). When considering top versus bottom responders, lower connectivity is associated with better cognitive outcomes in PD while higher connectivity is associated with better cognitive outcomes in AD (Figure S4 in supporting information). Additionally, both PD and AD showed weak non‐significant negative correlations between baseline cognition and post‐DBS cognitive outcome. There was no difference between the two correlations when comparing results between PD and AD (Figure S5 in supporting information).

To evaluate if this result was unique to the PD discovery cohort,^8^ we analyzed an independent cohort of PD patients who received DBS to the STN (PD validation cohort, *n* = 28, age 62 ± 7). Higher connectivity between STN DBS sites and the subiculum was again associated with poor cognitive outcomes in PD, which was significantly different from the association with improved outcomes in the AD cohort (ΔR = –0.44, *p* = 0.043). We found no significant differences between stimulation sites of the PD discovery (*x* = ± 12.9 ± 2.0 mm, *y* = –13.2 ± 2.2 mm, *z* = –4.9 ± 2.8 mm) and PD validation (*x* = ± 10.6 ± 1.5, *y* = –12.6 ± 2.6, *z* = –6.2 ± 2.4) cohorts in the *x* (*t* = –1.8, *p* = 0.070), *y* (*t* = –1.4, *p* = 0.16), nor *z* axes (*t *= 1.7, *p* = 0.088), whether mirroring stimulation sites or considering each side independently.

### 3.2 Age determines impact of subiculum connectivity on cognition across PD and AD

Next, we investigated whether differences in age might explain the paradoxical difference in cognitive outcomes. Given prior literature showing the effects of DBS on cognition can be influenced by age,^11^, ^14^, ^15^, ^23^, ^25^ we tested whether different ages could be driving the different cognitive outcomes reported in PD and AD (Figure 3D–I). We split both PD and AD patients into older and younger subgroups by age 65, based on prior literature suggesting differences in post‐DBS cognitive outcomes in patients above and below age 65.^25^, ^53^

Across all three DBS cohorts, we found that DBS sites more connected to the subiculum were associated with improved cognitive outcomes in older patients (*r* = 0.36, *p* = 0.019), consistent with results in the full AD cohort. Conversely, DBS sites more connected to the subiculum were associated with worse cognitive outcomes in younger patients (*r* = –0.31, *p* = 0.032), consistent with results in the PD cohorts. This difference in correlation between age‐based subgroups was larger than expected by chance (ΔR = 0.67, *p* = 0.0091) and was significant between age groups within each cohort individually, including the PD discovery cohort (ΔR = 0.80, *p* = 0.037), the AD cohort (ΔR = 0.55, *p* = 0.0073), and the PD validation cohort (ΔR = 0.65, *p* = 0.029).

These opposite age‐based effects were present when splitting patients at age 70, which has been suggested as relevant to post‐DBS cognitive decline in PD.^44^, ^45^ The above results were also significant regardless of using Spearman correlation or Pearson correlation, and were replicable in the PD validation cohort. The results were also unchanged when using a parametric test (analysis of covariance), and independent of whether we used a mixed effects or a fixed effects analysis to account for cohort or disease (Supplementary Materials 1.5.1, Figure S6 in supporting information).

### 3.3 Age, subiculum connectivity, and cognition interact similarly across PD and AD

Rather than split our cohort into subgroups based on an age cutoff, we next used linear modeling to test whether age (treated as a continuous variable) would influence the effect of subiculum connectivity on cognitive outcomes. Across all cohorts, we found a significant interaction between age and subiculum connectivity in estimating cognitive outcomes (β_interaction _= 0.25, *p* = 0.0007). This result was specific to these three variables, robust to folded validation, robust to different statistical models, independent of whether we use partial correlation or linear models, and independent of whether we used connectivity to the whole‐brain memory network^54^ or our subiculum region of interest. Details of the above control analyses are available for the PD cohorts (Supplementary Materials 1.5.2 in supporting information), and the AD cohort (Supplementary Materials 1.5.3 in supporting information).

**FIGURE 4:**
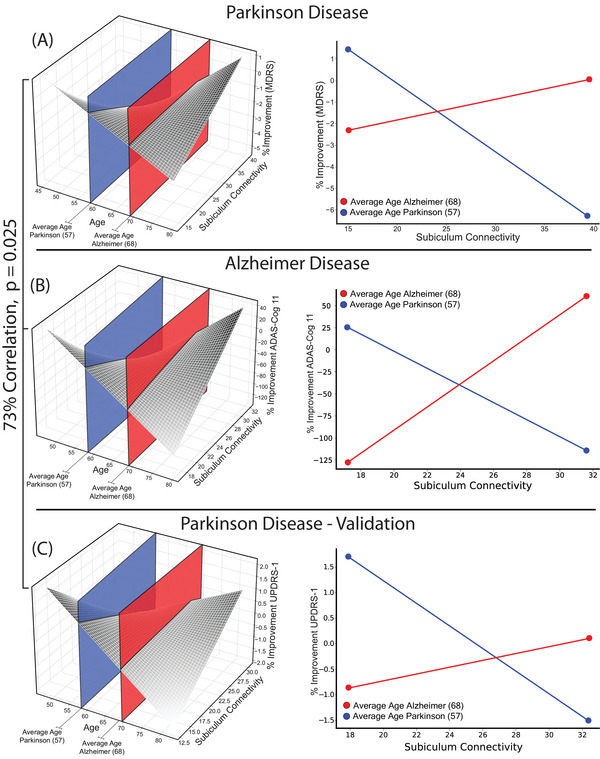
Across PD and AD, age similarly moderates effect of subiculum connectivity on cognitive outcomes. Left, Surface plots showing cognitive outcome as a function of age and subiculum connectivity for PD discovery (A), AD (B), and the PD validation cohorts (C). Surface plots showed similar topography across the three cohorts (73% spatial correlation, *p* = 0.025). Right, The blue and red planes from each plot, but flattened into lines for visualization. These demonstrate how subiculum connectivity relates to cognitive outcomes, estimated at age 57 (blue) and 68 (red) for each of the three DBS cohorts. AD, Alzheimer's disease; DBS, deep brain stimulation; PD, Parkinson's disease.

We plotted cognitive outcomes as a linear function of age and subiculum connectivity for each of the three DBS cohorts (Figure 4), and found these three cohorts behaved more similarly than expected by chance (spatial *r* = 0.73, *p* = 0.025). The AD distribution was similar to both the PD discovery (spatial *r* = 0.93, *p* = 0.028) and PD validation cohorts (spatial *r* = 0.92, *p* = 0.045). Goodness of fit testing demonstrated that each cohort's linear model could be used to predict cognitive outcomes in the independent cohorts (min *F* = 5.8, max *p*_FWE_ = 0.012), including cross‐prediction of cognitive outcomes between PD and AD (min *F *= 12.8, max *p*_FWE_ = 0.0008).

### 3.4 The interaction with subiculum connectivity is specific to age and cognition

We next investigated if other demographic factors could be influencing cognitive outcomes in PD and AD. Given prior reports of cognitive baseline being a critical factor in post‐DBS cognitive outcomes, we investigated how baseline cognitive function relates to cognitive outcomes across our cohorts. We repeated the above analyses, using cognitive baseline in place of age. We found no significant correlation between cognitive baseline in PD (*r* = –0.12, *p* = 0.52), nor AD (*r* = 0.24, *p* = 0.11). Cognitive baseline did not significantly interact with subiculum connectivity overall (β_interaction _= 0.1, *p* = 0.65), nor within the individual cohorts. Further, the influence of cognitive baseline on subiculum connectivity was not similar across PD nor AD cohorts (spatial *r* = –0.0097, *p* = 0.091). These cohorts could not cross‐predict each other (*p* = 0.78). Baseline cognition was largely unrelated to outcomes across various statistical models, folded validations, controlled variables, and analyses (Supplementary Materials 1.5.4 in supporting information).

We also tested if sex could be influencing the cognitive outcomes but found no direct relation between sex and cognitive outcomes (β = 0.18, *p* = 0.45), nor a moderating effect of sex upon factors like cognitive baseline (β_interaction_ = –0.005, *p* = 0.99), subiculum connectivity (β_interaction_ = –0.01, *p* = 0.96), nor age (β_interaction_ = –0.22, *p* = 0.36), whether controlling for cohort in fixed effects or mixed effects models.

The above results were also specific to connectivity to the subiculum (vs. six other regions of interest, Supplementary Materials 1.5.5 in supporting information). Although our primary analyses focused on connectivity between VTAs and the subiculum, this is unlikely to be the only connection important for DBS‐induced cognitive changes. We therefore repeated our analyses, examining connectivity between VTAs and all brain voxels (Supplementary Materials 1.5.6 in supporting information). We identified all connections that co‐varied with cognitive changes (Figure S7 in supporting information) as well as connections that showed an interaction among age, connectivity, and cognitive outcome (Figure S8A in supporting information). Results of these whole‐brain analyses align with our results focused on the subiculum, as the peak interaction between age, connectivity, and cognitive outcome was in the subiculum (Figure S8B). The whole‐brain interactions among age, connectivity, and cognitive outcomes were similar to the subiculum's connectivity profile across both PD and AD (Figure S9 in supporting information), and the voxels sharing significant interactions across PD and AD fell exclusively within the subiculum's connectivity profile (Figure S10 in supporting information).

### 3.5 Deriving the inflection point in age

We next wondered at which age subiculum connectivity flips from being associated with worse cognitive outcomes to better cognitive outcomes. To investigate, we began by deriving the inflection points in age for each dataset (Supplementary Materials 1.6, Figure S11 in supporting information). These age inflection points were remarkably similar across the three DBS datasets: 64.7 years in PD, 65.3 years in AD, and 66.9 years in our PD validation cohort, with an average inflection of 65.6 years. The similarity in inflections was present despite significant differences across the cohorts (Figure S12 in supporting information), including the age means (*p*_Max_ = 0.0001) and age distributions (*p*_Max_ = 0.037), and was not due to difference in connectivity (Figure S13 in supporting information). We repeated our above age‐based subgroup analyses using these data‐driven inflection points (ages 64.7 in PD and 65.3 in AD). The results were unchanged and the difference in correlation between age‐based subgroups was larger than expected by chance (ΔR = 0.67, *p* = 0.0091). The age‐based differences were significant within each cohort individually, including the PD discovery cohort (ΔR = 0.80, *p* = 0.037), the AD cohort (ΔR = 0.55, *p* = 0.0073), and the PD validation cohort (ΔR = 0.65, *p* = 0.029).

### 3.6 Deriving the inflection point in subiculum connectivity

Having considered the data‐driven inflection points in age, we next considered the data‐driven inflection points in subiculum connectivity: the connectivity value at which older age inflects from being associated with cognitive impairment to cognitive improvement (Supplementary Materials 1.7.1 in supporting information). The data‐driven inflection point for connectivity was a *t* value of 24.2 in our PD discovery cohort, 23.8 in our AD discovery cohort, and 24.0 in our PD validation cohort. These inflection points were again similar across cohorts despite large differences in each cohort's connectivity (Supplementary Materials 1.7.2, Figure S14 in supporting information), were not driven by subgroup effects (Figure S15 in supporting information), and can be used to create connectivity‐based subgroups which can reproduce the subgroup‐based analyses from Figure 2 (Supplementary Materials 1.7.3, Figures S16, S17 in supporting information).

### 3.7 Combining age and connectivity inflections reveals top‐performing groups across PD and AD

**FIGURE 5:**
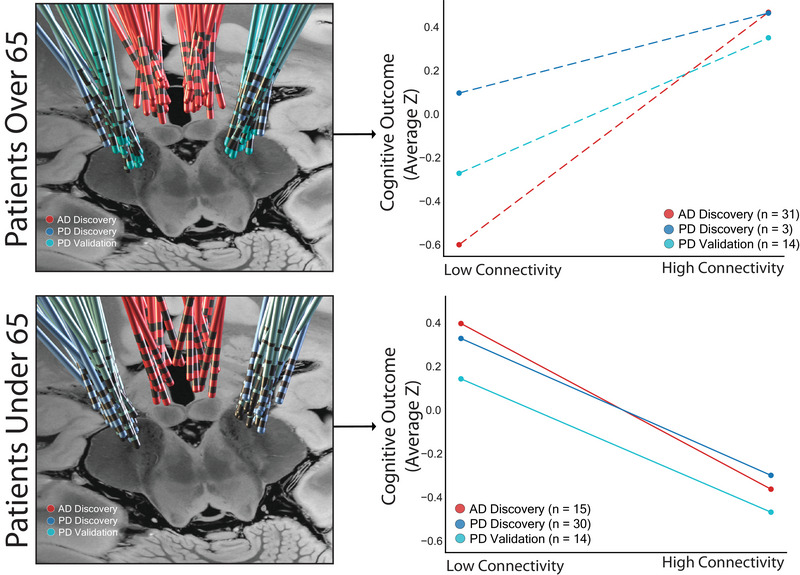
Cognitive effects of DBS are similar across cohorts when binned by age and connectivity. Left, Electrode locations from each age‐based subgroup across all three DBS cohorts. Right, Plot of average cognitive outcomes in each DBS cohort, binned by age and connectivity of their DBS sites to the subiculum. Older patients with high connectivity had better cognitive outcomes than those with low connectivity (*p* = 0.023). Conversely, younger patients with low connectivity had better cognitive outcomes than those with high connectivity measured by contrast testing (*p* = 0.0083). Results were similar across all three DBS cohorts (colored lines). AD, Alzheimer's disease; DBS, deep brain stimulation; PD, Parkinson's disease.

**FIGURE 6:**
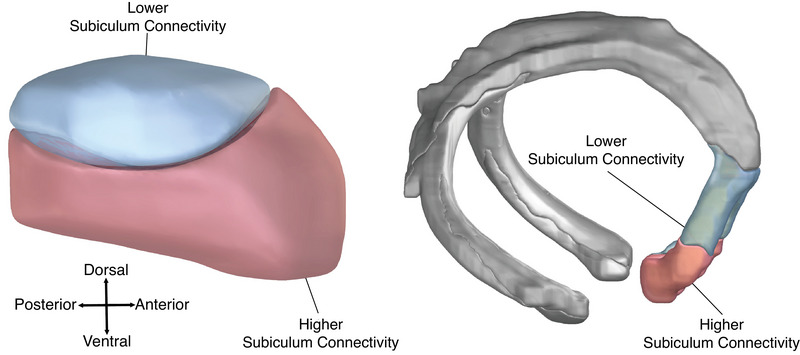
Schematic illustration of age‐based stimulation targets. Anatomical renders of the STN (left) and the fornix (right). Blue regions represent locations within the STN and fornix which have subiculum connectivity below the inflection point (*t* < 24.2), while red regions represent locations which have subiculum connectivity above the inflection point (*t* > 24.2). Older patients with DBS sites in regions above the connectivity inflection point had better outcomes than other older patients with DBS sites below the connectivity inflection (planned contrast, *p* = 0.023). Younger patients with DBS sites in regions below the connectivity inflection point had better outcomes than other younger patients with DBS sites above the connectivity inflection (planned contrast, *p* = 0.0083). DBS, deep brain stimulation; STN, subthalamic nucleus.

Using the age and connectivity inflection points together allows for identification of four subgroups in each cohort (e.g., young patients with low DBS site subiculum connectivity). We compared average cognitive outcomes within these subgroups (Figure 5), and found older patients had better outcomes when DBS sites were above the connectivity inflection point (planned contrast, *p* = 0.023), while young patients had better outcomes when DBS sites were below the connectivity inflection points (planned contrast, *p* = 0.0083). The interaction between age and connectivity was again present (*p* = 0.012), and was robust regardless of model choice, mixed effects, or fixed effects analysis (Supplementary Materials 1.8.1, Figure S18 in supporting information). This finding was unchanged when using literature‐based cutoffs (age 65 or 70). We next generated a schematic illustration showing voxels within the STN and fornix that showed high versus low connectivity to the subiculum, using our data‐driven inflection point as a cutoff (Figure 6).

### 3.8 Hippocampal volume mediates the interaction of age with connectivity

Although age may be a useful clinical metric to select patients for DBS,^24^, ^25^ age might be a proxy of an underlying pathophysiologic process. Given known relationships between age and hippocampal volume,^55^, ^56^ we tested for a relationship in our cohorts. We found increasing age was indeed negatively correlated with hippocampal gray matter volume across our three DBS cohorts (Pearson *r* = –0.38, *p* = 0.001).

We next investigated if the interaction between age and subiculum connectivity was mediated by hippocampal volume loss. We found a significant mediation (*p* = 0.0317). This was a full mediation, as the previously significant age–connectivity interaction (*p* = 0.012) became non‐significant (*p* = 0.28) when including hippocampal gray matter volume in the regression. This full mediation was also present when run on PD and AD cohorts separately, or when controlling for cohort in the overall mediation (Supplementary Materials 1.9.1 in supporting information). This mediation was also specific to hippocampal volume, as mediation was not seen using parahippocampal gyrus volume (*p* = 0.33) nor entorhinal cortex volume (*p* = 0.25). The mediation was present whether using the left (*p* = 0.0098), right (*p* = 0.044), or average hippocampal volume (*p* = 0.0317).

Finally, having identified several variables that contribute to variance in DBS cognitive outcomes, we examine how much variance might be explained. We found that age, connectivity, and hippocampal volume each explained only modest variance when considered independently (Supplementary Materials 1.9.1, Table S2 in supporting information) but explained significant variance when considered together (*R* ^2^ = 0.33, *p* = 0.032).

## 4 DISCUSSION

### 4.1 Interpretation of results

We find the different cognitive impact of DBS in AD and PD, which first seem paradoxically opposed, were driven by differences in subiculum connectivity, patient age distributions, and patient hippocampal volumes. When these factors are considered, both diseases demonstrate similar cognitive responses to DBS.

### 4.2 Resolving an existing paradox

Recent papers suggest that DBS electrodes functionally connected to the hippocampus impair cognition in PD^8^ but improve cognition in AD.^21^ We showed that this paradoxical observation is likely due to differences in patient age and hippocampal volume. Because PD DBS patients tend to be younger and have relatively preserved hippocampal volume, DBS sites connected to the subiculum are associated with poor cognitive outcomes. Because AD DBS patients tend to be older and have low hippocampal volume, DBS sites connected to the subiculum tend to be beneficial.^21^ When we separate PD and AD patients into age‐based subgroups, the paradox disappears, and we see similar results in both disorders, but with opposite results in each age‐based subgroup.

### 4.3 Age, atrophy, and DBS

While our results and previous literature have implicated age as an important process influencing DBS cognitive outcomes,^17^ it is likely that age is just a proxy of the underlying true physiologic process. Our results suggest hippocampal volume may be a useful imaging‐based biomarker of this physiological process, as it fully mediated our observed interaction of age with subiculum connectivity on cognition. This result is consistent with prior work suggesting that hippocampal volume can change after DBS. Specifically, hippocampal volumes have been observed to increase in top‐responding older AD DBS patients,^57^ but decrease in younger PD STN DBS patients.^58^

Of all brain regions, age‐related atrophy tends to be particularly prominent in the hippocampus.^56^, ^59^, ^60^, ^61^ Within the hippocampus, the subiculum is most susceptible to age‐related atrophy,^62^, ^63^ with its integrity being highly related to cognitive dysfunction in AD,^61^, ^64^, ^65^, ^66^, ^67^ PD,^68^, ^69^, ^70^, ^71^ and even in older patients without a neurodegenerative diagnosis.^72^, ^73^, ^74^ However, this correlation between hippocampal volume and cognitive dysfunction is only robustly observed after age 65,^75^, ^76^ the age at which hippocampal atrophy begins to rapidly accelerate.^55^, ^77^ The mechanism of this acceleration of hippocampal atrophy at age 65 remains unclear.^78^ It may be related to an age‐dependent cascade of cellular dysfunction,^79^, ^80^ neuronal hyperactivity,^81^, ^82^, ^83^, ^84^, ^85^, ^86^, ^87^, ^88^ epileptiform discharges,^86^, ^89^, ^90^, ^91^ and dendritic pruning,^86^, ^92^ all of which combine to result in atrophy.^81^, ^86^, ^93^ In animal models, intervening on this cascade can normalize hippocampal hyperexcitability,^94^, ^95^, ^96^, ^97^, ^98^, ^99^ restore hippocampal volume,^60^, ^97^, ^100^, ^101^, ^102^ and improve cognitive function.^94^, ^97^, ^98^, ^99^, ^103^

It is possible that high‐frequency DBS sites connected to the hippocampus also disrupt this cascade, resulting in beneficial cognitive effects in patients older than 65 or those with hippocampal volume loss. The hypothesis that high‐frequency DBS improves cognition through disruption of pathological hyperactivity is consistent with proposed mechanisms for DBS improvements in tremor, PD, and epilepsy.^104^, ^105^, ^106^ The hypothesis that DBS works through remote effects on connected brain regions is consistent with remote changes in metabolism,^107^, ^108^ gray matter volume,^106^, ^109^ or neurotransmitter release^110^ distal from DBS sites, as well as numerous studies relating DBS connectivity to clinical outcomes.^8^, ^20^, ^26^, ^111^ Finally, the hypothesis that normalizing hippocampal hyperactivity can lead to improved cognition in patients is consistent with reported benefits of anti‐epileptic medication in mild cognitive impairment.^99^

In patients younger than 65, in whom hippocampal volume is relatively stable,^55^ DBS sites connected to the hippocampus may disrupt more physiological activity than pathological activity, resulting in cognitive impairment. This hypothesis is consistent with lesion data, in which patients under the age of 65 that had strokes connected to the subiculum developed memory impairment.^36^ Collectively, our results suggest that DBS to an intact memory circuit (in younger patients with normal hippocampal volume) may result in deleterious cognitive effects, while DBS to a malfunctioning memory circuit (in older patients with lower hippocampal volumes) may result in better cognitive outcomes. It also remains to be seen if this effect is unique to cognition, or if it has relevance other symptoms in which the effect of DBS may change as a function of time or age. For example, DBS for essential tremor can become less effective over time, which has been linked to increased atrophy.^112^ Future studies are needed to understand how age, time, and atrophy may influence the effect of DBS on other symptoms.

### 4.4 Clinical implications

Our results may have some clinical implications. We investigated the effects of baseline cognition, sex, and age in both PD and AD. In PD, poor baseline cognition^9^, ^10^, ^17^, ^25^, ^113^ is a risk for cognitive decline after STN DBS, which has contributed to clinical guidelines cautioning against the use of DBS in older PD patients, although we did not see this relationship reproduced.^9^, ^10^, ^11^, ^15^, ^17^, ^21^, ^25^, ^113^ While sex has also been reported as a risk factor for post‐DBS cognitive decline in PD,^114^, ^115^, ^116^ we did not find a significant relationship between sex and post‐DBS cognitive outcomes in PD nor AD. Prior studies investigating age have not found a clear association between age and post‐DBS cognitive decline.^11^, ^15^, ^21^ However, our results do suggest patient age may be valuable in counseling patients regarding the expected better cognitive outcomes (or cognitive side effects) of DBS.

Regarding PD patients, our results do not support excluding PD patients from DBS based on older age. In fact, if one selects a DBS contact with high subiculum connectivity, older patients with PD could potentially get better cognitive outcomes from DBS. Although not directly tested in this study, a provocative hypothesis based on our data is that the ideal stimulation site for cognition might change as the patient ages. A PD patient implanted at age 50, but who has now turned 70, could theoretically benefit from DBS reprogramming to a site that is more connected to the subiculum, resulting in improved cognition. Such a hypothesis remains to be tested in a clinical trial.

With regard to AD, age is being used to guide clinical trials by excluding patients under the age of 65.^17^, ^25^ This decision was made after it was observed that younger patients tended to do worse than older patients.^25^ Our results provide data‐driven support for this decision, assuming the goal is to target the fornix and maximize connectivity to the hippocampus. In patients over age 65.3, our results support choosing the electrode contact with maximum connectivity to the subiculum of the hippocampus.

With regard to DBS targets for cognition, it remains unclear if the STN could be a stimulation site for AD, or cognition generally. Although our results suggest that both the STN and fornix DBS sites influence cognition, and may do so through connectivity to the subiculum, this does not mean that these DBS targets are equivalent. For example, patients occasionally report memory flashbacks during fornix stimulation, but such phenomena have not been observed with STN DBS. One possibility is that flashbacks are mediated via a different neuroanatomical pathway, one that is not shared with the STN.^20^ Another possibility is that the fornix and STN influence the subiculum, but do so in different ways that result in different subjective experiences. Further work is needed to explore these differences.

### 4.5 Limitations

There are several limitations regarding this work. First all data analyses were retrospective, and hypotheses generated by this work remain to be tested prospectively to understand causality. Further, we are combining DBS data from multiple studies, multiple diseases, and different cognitive outcome measures. This introduces considerable heterogeneity which may bias us against identification of consistent finds across our different DBS cohorts.^117^

Second, another limitation of our study is that different cognitive tests bridging multiple cognitive domains were used across our different DBS cohorts. Assessment of cognitive changes after DBS may be heavily influenced by the choice of tests used to measure cognition. However, this variability in cognitive tests should have biased us against the present findings, namely identification of a consistent neuroanatomical substrate for DBS‐induced cognitive changes across independent DBS cohorts.

Third, there are many variables that contribute to variance in cognitive outcome after DBS,^116^, ^118^, ^119^ thus the variance explained by any individual variable is small. We show that DBS site (and site connectivity) explains a small amount of variance, age explains a small amount of variance, and hippocampal volume explains a small amount of variance. As these variables are combined, explained variance increases (see Table S2). Future work evaluating additional relevant variables will be needed to build prognostic models of cognitive outcomes after DBS.

Fourth, there is potential for registration inaccuracies in the location of the DBS electrodes, especially in the context of brain atrophy. We used advanced techniques for localizing electrodes, including brain shift correction,^31^ multispectral normalization,^32^ phantom validated electrode localizations,^120^ and manual refinement of atrophy with warp fields.^33^ However given that atrophy co‐varies with age, it is possible that systematic errors in electrode registration could occur. Similarly, registering all patients to a common brain atlas may obscure important differences in patient‐specific anatomy or atrophy.^121^ Our analysis of hippocampal volume (measured in each individual patient) speaks to the importance of these individual differences and highlights the value of combining atlas‐based analyses with analyses in patient space.

Fifth, we are limited by access to additional cohorts. Of the roughly 60 patients who have received DBS for AD worldwide, we analyzed 46 which were in the largest randomized controlled trial.^122^ Other studies are observational, underpowered, or less rigorously controlled, complicating meaningful analysis. Thus, it is challenging to test additional stimulation sites, cohorts, or stimulation parameters, especially in AD.

Last, our connectivity analyses are based on normative functional connectivity data, using connectivity data from a large cohort of healthy participants, not individual patients.^38^ This analysis approach has yielded robust findings in other DBS network studies,^8^, ^20^, ^26^, ^111^, ^123^ and prior work suggests that using disease‐specific or patient‐specific connectomes yield similar results.^107^, ^111^, ^124^ However, as the quality of patient‐specific connectivity imaging improves, analyses leveraging patient‐specific connectivity may yield additional insights. Such future work might also test whether connectivity between the DBS site and subiculum changes from pre‐ to post‐operatively, similar to prior studies of hippocampal volume.^57^, ^58^

## CONFLICT OF INTEREST STATEMENT

The authors have no conflicts to disclose. Author disclosures are available in the supporting information.

## CONSENT STATEMENT

This study was conducted in accordance with ethical standards and approved by the institutional review board (IRB) of the Brigham and Women's Hospital and Harvard Medical School, Boston, Massachusetts. Given the secondary use of research data, the study was exempted from obtaining informed consent. The discovery AD cohorts includes data in compliance with the IRBs across seven international centers included in the ADvance trial (NCT01608061),^25^ and the Toronto‐based pilot trial (NCT00658125).^18^ The discovery PD cohort is published in compliance with the IRB of Würzburg University Hospital and Beth Israel Deaconess Medical Center, Boston, USA (IRB Protocol no. 2018P000128). The validation of the PD cohort was conducted in compliance with the IRB of Israel Deaconess Medical Center, Boston, USA (IRB Protocol no. 2015P00028).

## Supporting information



Supporting Information

Supporting Information
