# Supplementary material for: Cognitive outcomes of deep brain stimulation depend on age and hippocampal connectivity in Parkinson's and Alzheimer's disease
Source: Alzheimers Dement. 2025 Aug 21;21(8):e70498. doi: 10.1002/alz.70498 (PMC12368798; doi:10.1002/alz.70498)
Supplement: Supplementary file 1 — Supporting Information [file ALZ-21-e70498-s001.docx]

**Supplementary Figure 1. One year percent change in cognitive outcomes in PD and AD after DBS.** A) Strip plot of percent cognitive change in PD. Five patients showed positive changes, suggesting some degree of cognitive improvement. B) Strip plot of percent cognitive change in AD. Nine patients showed positive changes, suggesting some degree of cognitive improvement. The MDRS ranges from 0 to 144. The ADAS-Cog scale ranges from 0 to 70.

- 1. Supplementary Methods

The patient electrodes are first localized and reconstructed within MNI space (Supplementary Figure 2). Subsequently, the electrical field delivered to the deep brain stimulator is estimated, and the VTA is derived from this. The average blood-oxygen level dependent signal within this VTA, across 1000 healthy controls, is then correlated to the average blood-oxygen level dependent signal within the subiculum of the same controls. This provides the connectivity of each patient’s VTA to the subiculum.

Supplementary Figure 2. Methods used to estimate DBS electrode connectivity to the subiculum. Top) Parkinson Disease. Bottom) Alzheimer Disease. Left) Reconstruction of DBS electrodes in MNI space, followed by estimation of the electrical fields delivered by each DBS electrode. The VTA is then estimated from the electrical field. The VTA is then ROI-ROI correlated with the subiculum using a connectome of 1000 healthy controls. This provides the subiculum connectivity value.

1.2 Derivation of the Inflection Point in the Response Topology

An algebraic description of a phenomenon can be solved for various properties such as inflection points in variables. The multivariate regression provides an algebraic description how age and subiculum connectivity relate to cognitive outcomes (Supplementary Equation 1). Thus, it can be solved to identify the inflection points. This is accomplished by taking the partial derivative of cognitive outcomes with respect to age and subiculum connectivity (Supplementary Equation 1). This sets up a system of equations which can be simultaneously solved to identify the corresponding values of each variable when an inflection occurs (Supplementary Equation 1).

For visualization of the partial derivatives across each variable, the standard approach is to calculate the gradient of change in the predicted variable as a single coefficient changes, while holding all other coefficients stable. This is done with standard packages such as NumPy. The gradient can then be directly visualized.

$$Outcomes=\beta_{1}Age+\beta_{2}SBC+\beta_{3}(Age*SBC)$$

Supplementary Equation 1. Multivariate equation relating age and subiculum-retrosplenial complex connectivity to cognitive outcomes.

$\frac{d Outcomes}{d Age}=\beta_{1}Age+\beta_{3}SBC$

$\frac{d Outcomes}{d SBC}=\beta_{2}SBC+\beta_{3}Age$

Supplementary Equations 2. Partial derivatives of outcomes with respect to age and subiculum-retrosplenial complex connectivity.

$-\frac{\beta_{1}}{\beta_{3}}=SBC$

$-\frac{\beta_{2}}{\beta_{3}}=Age$

Supplementary Equations 3. Partial derivatives solved for critical point of age and subiculum-retrosplenial complex activity.

1.3 Sensitivity Analysis of Shared Topology

In addition to identification of the whole-brain peak maximally associated with cognitive outcomes, the topology of the cognitive outcomes was itself assessed. After finding the whole-brain connections correlated to cognitive decline in PD and AD, we investigated their microanatomy. Not only were the maxima one millimeter apart, but they fall within the same histologically defined Brodmann Area (Supplementary Table 1).

Next, the cognitive correlation maxima derived from one disease could be used as an ROI. We repeated our original analysis, and derived the connectivity of each VTA from one disease to the other disease’s maxima. Connectivity of PD VTAs to the AD cognitive improvement peak was again correlated with cognitive decline in PD (Pearson R = -0.39, p < 0.05). Conversely, connectivity of the AD VTAs to the PD cognitive decline peak was again correlated to cognitive improvement (Pearson R = 0.45, p < 0.05).

Supplementary Table 1. Locations of Local Maxima Within Memory Region of Interest.


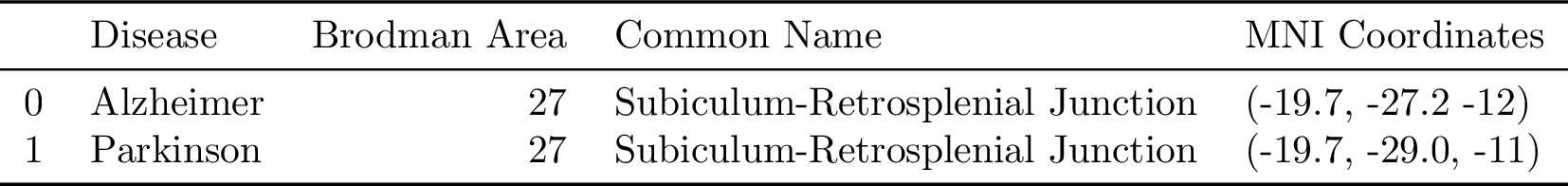


1.4 Sensitivity Analyses for Validation of Paradox

Subiculum connectivity was related to cognitive outcomes using Spearman correlation. We related both ROI (VTA & subiculum) and whole-brain (VTA & subiculum connectivity profiles) to cognitive outcomes. Subiculum connectivity and the spatial correlation of patient VTA connectivity profiles to subiculum connectivity profiles were related to cognition. PD cognitive outcomes were negatively but not significantly correlated to subiculum connectivity (Spearman Rho = -0.27, p > 0.05). Spatial correlation to the subiculum connectivity profile was significantly negatively correlated with PD cognitive outcomes (Spearman Rho = -0.44, p < 0.05). AD was the opposite, with subiculum connected DBS being positively correlated with cognitive outcomes (Spearman Rho = 0.33, p < 0.05). AD spatial correlation to the subiculum connectivity profile was also positively correlated to cognitive outcomes (Spearman Rho = 0.31, p < 0.05). There was a significant difference between how PD and AD cognitive outcomes correlated to subiculum connectivity (ΔRho = -0.60, p < 0.05). This was also true for spatial correlation of VTA connectivity profiles to the subiculum connectivity (ΔRho = -0.75, p < 0.05). There was no difference within diseases across connectivity metrics (PD: ΔRho = 0.17, p > 0.05; AD: ΔRho = 0.29, p > 0.05).

Results were then repeated with Pearson correlation (Supplementary Figure 3). PD cognitive outcomes were negatively but not significantly correlated to subiculum connectivity (Pearson R = -0.26, p > 0.05). Spatial correlation to the subiculum connectivity profile was significantly negatively correlated with PD cognitive outcomes (Pearson R = -0.38, p < 0.001). AD was the opposite, with subiculum connected DBS being positively correlated with cognitive outcomes (Pearson R = 0.31, p < 0.05). AD spatial correlation to the subiculum connectivity profile was also positively correlated to cognitive outcomes (Pearson R = 0.33, p < 0.05). There was a significant difference between how PD and AD cognitive outcomes correlated to subiculum connectivity (ΔR = -0.55, p < 0.05). This was also true for spatial correlation of VTA connectivity profiles to the subiculum connectivity (ΔR = -0.51, p < 0.05). There was no difference within diseases across connectivity metrics (PD: ΔR = 0.13, p > 0.05; AD: ΔR = 0.03, p > 0.05).


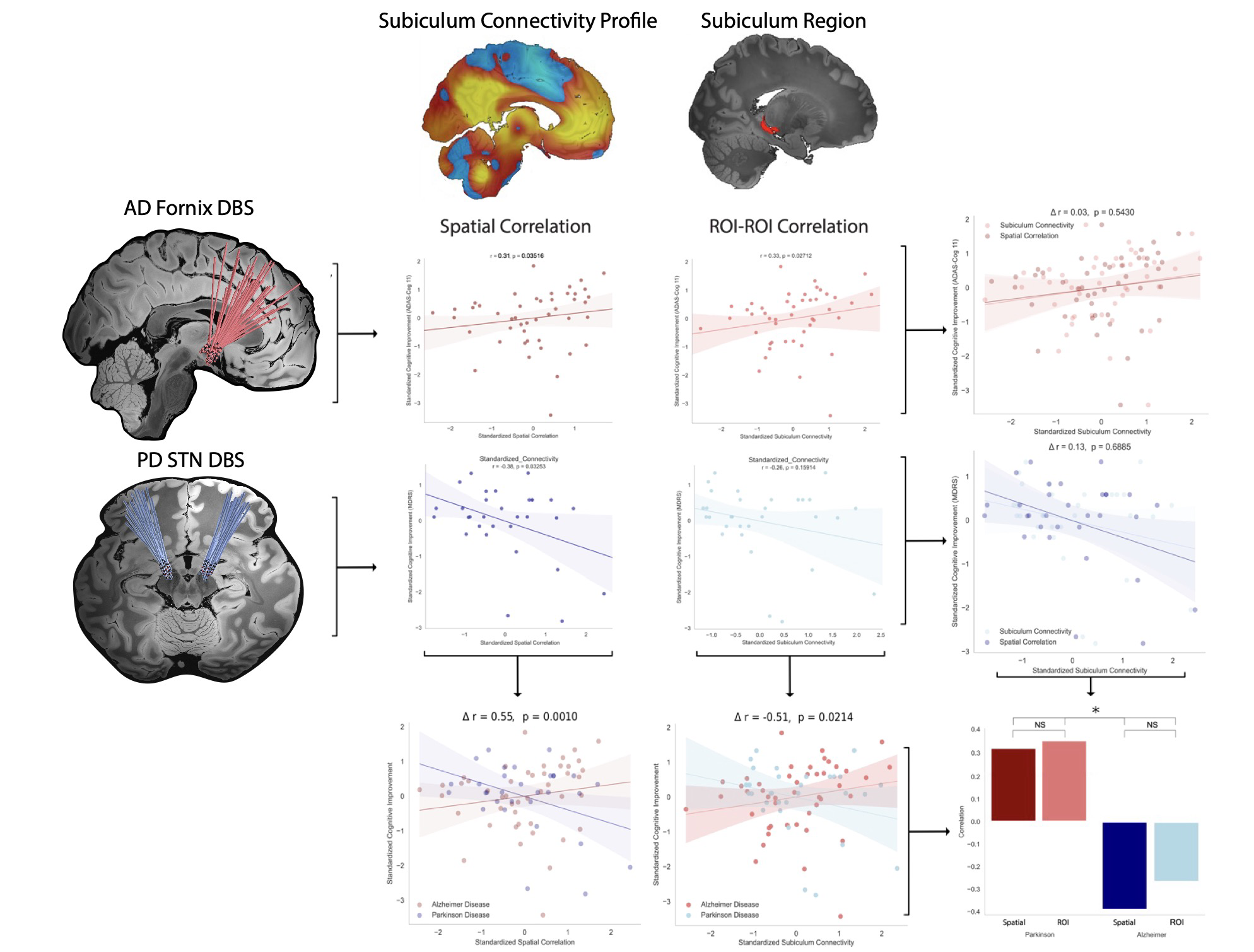


Supplementary Figure 3. Diagrammatic illustration of delta-correlation experiment using Spearman Correlation. PD and AD cohorts respond significantly differently to both subiculum connectivity (ΔR = 0.51, p = 0.0214) and connectivity to the entire memory network (ΔR = 0.55, p = 0.0010). There is no significant difference in how the AD cohort responds to subiculum ROI connectivity versus memory network connectivity (ΔR = 0.03, p = 0.54), nor in how the PD cohort responds to subiculum ROI versus memory network connectivity (ΔR = 0.13, p = 0.6885).


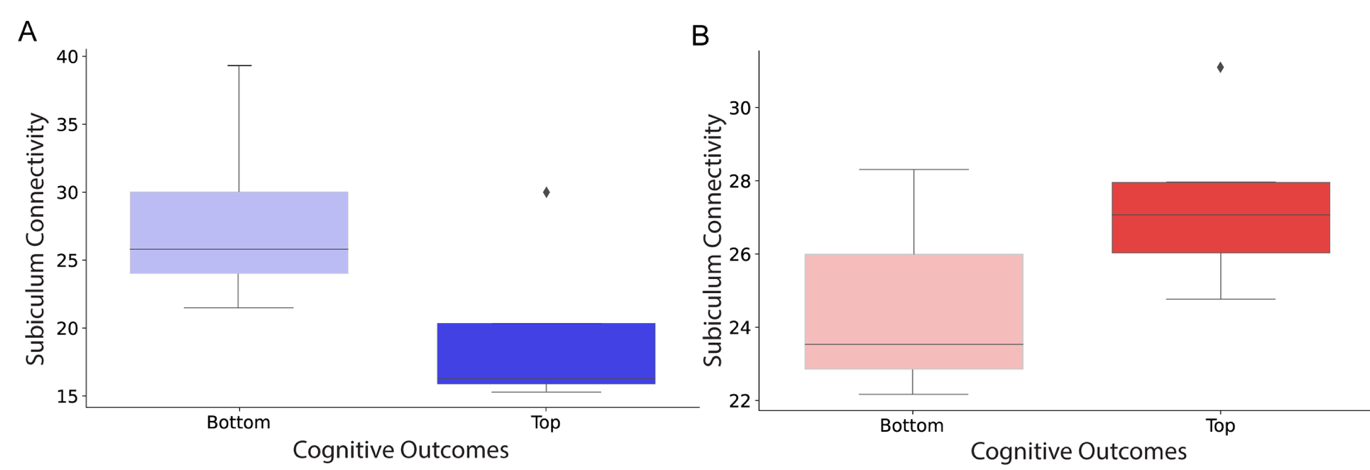
Supplementary Figure 4. Subiculum connectivity of top versus bottom responders. A) The subiculum connectivity cognitively improved PD patients (n = 5) versus sample-matched bottom responders (n = 5). Cognitively improved patients had lower subiculum connectivity (U = 3.94, p = 0.047). B) The subiculum connectivity of cognitively improved AD patients (n = 9) versus sample-matched bottom responders (n = 9) in AD. Cognitively improved patients have higher subiculum connectivity (U = 4.34, p = 0.037).

Supplementary Figure 5. The paradoxical between AD and PD is not driven by inherent differences in how either disease responds to age or baseline cognition. A) Electrode locations for the PD (blue electrodes) and AD (red electrodes) discovery cohorts. Active contacts on each electrode are shown in red. B) Scatterplot showing that increased baseline cognitive status is associated with worse cognitive outcomes in PD (blue) and AD (red), with no significant difference between the two (ΔR = -0.13, p = 0.56). C) Boxplots demonstrating median and interquartile range of the bootstrapped correlations between subiculum connectivity and cognitive outcomes for PD (blue bar) and AD (red bar).

1.5.1 Parametric Evaluation of Age Group Effect on Cognition

Next, we categorized patients into age-based subgroups. We reproduced the original delta-R analysis with the validation cohort, finding the correlations of subiculum connectivity to cognitive outcomes were significantly more negative than in the AD (p < 0.05), but were not significantly different from the PD discovery cohort (p > 0.05).

We next performed an ANCOVA, controlling for the effect of connectivity and isolating the effect of the age subgroups upon cognitive outcomes (Supplementary Figure 6). We found a significant interaction between age group and connectivity (p < 0.05). We then plotted the average outcomes of age-optimized patients (e.g. young and low connectivity) to non-optimized patients (e.g. young and high connectivity). Using planned contrasts of the ANCOVA, we found young low-connectivity patients outperformed their high-connectivity counterparts (p < 0.05), as did the older patients with high connectivity compared to their low connectivity counterparts (p < 0.05). To visualize this, we created an estimated marginal means plot, which allows us to project the expected outcomes, ‘adjusted’ by the ANCOVA. For this, we projected the outcomes of patients exposed to high connectivity, then created a set of expected outcomes for each cohort, using both young and old patients. We again contrasted these age groups, finding a significant difference between how young and old patients were expected to perform in response to high connectivity (p < 0.05).

Further, these same results were reproduceable when using a mixed-effects model which more appropriately accounts for the variance inherent to the different cohorts (interaction p < 0.05). This interaction was stable whether using mixed effects, fixed effects, controlling for cohort as a covariate, or not controlling for cohort as a covariate.

After accounting for age, the average correlation of connectivity with outcomes became more robust. In the older patients, the average correlation was positive (average r = 0.48±0.12), while it was negative in the younger patients (average r = -0.40±0.18). The difference between these two groups was significant (Welch’s T-test, T = 7.05, p = 0.00305).


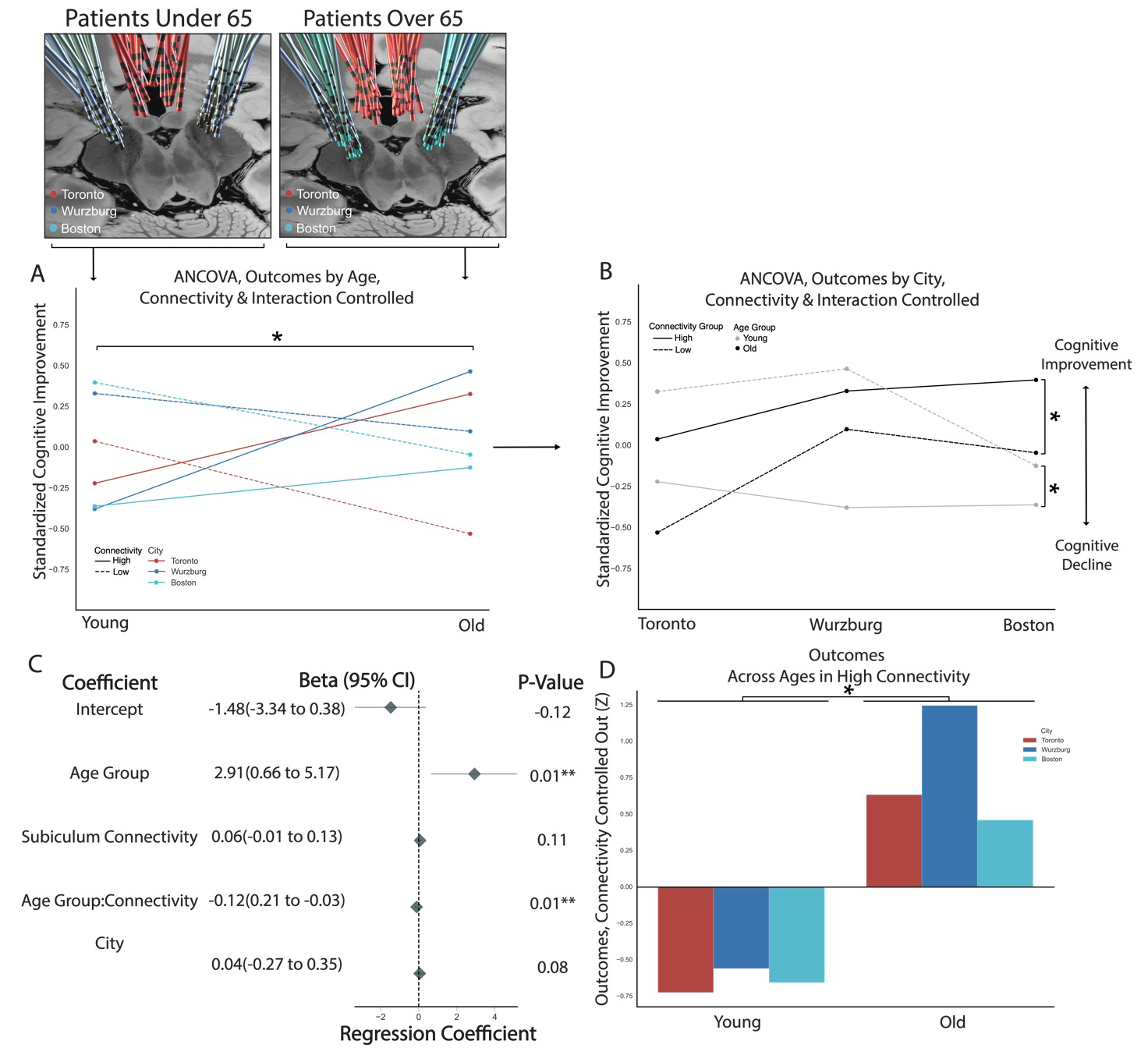


Supplementary Figure 6. ANCOVA demonstrates splitting by age inflection replicates the interaction found between connectivity and age. A) Interaction plot demonstrating average patient outcomes across each group. A significant interaction between age group and connectivity was detected by ANCOVA. B) Main effects plot, demonstrating the same average outcomes, but visualized across cohorts. The planned contrasts (derived from the ANCOVA) *within* age groups *across* connectivity levels are highlighted with significance bars. C) Forest plot representing the results of the ANCOVA. D) Estimated marginal means plot, projecting the situation wherein young and old patients from each cohort are exposed to high connectivity. This allows for ‘adjusting’ the effect of connectivity out, isolating how these patients are expected to respond. Contrast of these outcomes demonstrated a significant difference between age groups (p < 0.05). In A and B, patients are also split into connectivity subgroups; this is based on the inflection point in connectivity, and is done simply for eased visualization of the ANCOVA. The connectivity inflection does not influence the ANCOVA results. The connectivity inflection is discussed in the next section.

1.5.2 Sensitivity Analyses of Interaction Effect in Parkinson Disease

We found no correlation between the age variables and cognitive outcomes in either disease (R = 0.06, p > 0.05; Supplementary Figure 6A). Age was not correlated with either connectivity measure (Subiculum Connectivity: Pearson R = -0.06, p > 0.05; Spatial Correlation: Pearson R = -0.06, p > 0.05).

Next, we investigated the moderating effect of age upon subiculum connectivity, or their interaction. To do this, we used an OLS GLM. The model overall explained 19% of the variance in outcomes, equivalent to a Pearson correlation of 0.44, with a root mean squared error of 2.5% cognitive change (0.90 standard deviations). Hierarchical modelling was used to derive the additional explained variance attributable to the interaction effect with an F-test to compare the models (Additional R^2^ = 0.09, p >0.05). (Leave-one-out, R^2^=0.16 | 20-fold, R^2^=0.23 | 10-fold, R^2^=0.14 | 5-fold, R^2^=0.17 | 2-fold, R^2^=0.20 | 1-fold, R^2^=0.19).

To assess for robustness of the interaction effect, we tested if it persisted after controlling for other clinical covariates which may potentially confound the equation. The interaction effect of age with connectivity persisted after controlling for clinical covariates in the model (randomization status, baseline MDRS, UPDRS-3 motor score, p<0.05).

To evaluate the reliability of these findings without relying upon general linear models, we employed a model-free evaluation of this interaction. A partial correlation of the interaction term between age and subiculum connectivity was performed, finding a significant correlation with this term and outcomes even after accounting for the isolated contributions of age and subiculum connectivity (R_partial_ = 0.28, p < 0.05).

All analyses were reproduced using spatial correlation of each patient’s whole-brain VTA connectivity profile to the memory network.

1.5.3 Sensitivity Analyses of Interaction Effect in Alzheimer Disease

We found no correlation between the age variables and cognitive outcomes in either disease (R = 0.06, p > 0.05). Age was not correlated with either connectivity measure (subiculum: Pearson R = 0.04, p > 0.05; Spatial Correlation: Pearson R = 0.19, p > 0.05).

A general linear model regressed cognitive outcomes upon age, subiculum connectivity, and their interaction, finding a statistically significant interaction between age and subiculum connectivity (p < 0.05). The model overall explained 16% of the variance in outcomes, equivalent to a Pearson correlation of 0.40, with a root mean squared error of 24% cognitive change (0.85 standard deviations). Hierarchical modelling was used to derive the additional explained variance attributable to the interaction effect with an F-test to compare the models (Additional R^2^ = 0.135, p < 0.05). Cross validation was employed to demonstrating stability of this result (Leave-one-out, R^2^=0.15 | 20-fold, R^2^=0.15 | 10-fold, R^2^=0.16 | 5-fold, R^2^=0.17 | 2-fold, R^2^=0.20 | 1-fold, R^2^=0.16).

To assess for robustness of the interaction effect, we tested if it persisted after controlling for other clinical covariates which may potentially confound the equation. The interaction effect of age persisted after controlling for clinical covariates in the model (randomization status, baseline ADAS-Cog 11, baseline Clinical Dementia Rating Scale, p<0.05).

To evaluate the reliability of these findings without relying upon general linear models, we employed a model-free evaluation of this interaction. A partial correlation of the interaction term between age and subiculum connectivity was performed, finding a significant correlation with this term and outcomes even after accounting for the isolated contributions of age and subiculum connectivity (R_Partial_ = 0.35, p < 0.05).

All analyses were reproduced using spatial correlation of each patient’s whole-brain VTA connectivity profile to the memory network.

1.5.4 Analysis of Cognitive Baseline’s Impact on Cognitive Outcomes

We wondered if this interaction was specific to age, or if a more well-known demographic variable such as baseline cognition might better explain the paradox.^26,31^ Surprisingly, there was no interaction between baseline cognition and subiculum connectivity for PD (β_interaction_ = 0.16, p = 0.37) nor AD (β_interaction_ = 0.01, p = 0.94). Without the interaction, the relationship between lower baseline was associated with worse outcomes but was not significant in PD (r = -0.12, p = 0.52) nor AD (r = -0.24, p = 0.11).

Baseline cognition did not result in models that explained much variance in cognitive outcomes (PD: R^2^ = 0.06, AD: R^2^ = 0.04). Baseline cognition did not interact with subiculum connectivity upon cognitive outcomes in either disease (PD: p > 0.05; AD: p > 0.05), as evidenced by response topologies (Figure 4G, K). The joint distributions did not reflect a phenomenon that generalized well from one disease to the other, with low similarity of the joint distributions (Spatial Correlation = -0.0097, p > 0.05). Neither disease’s GLM was able to estimate outcomes of the other disease (PD GLM goodness-of-fit on AD, p > 0.05; AD GLM goodness-of-fit on PD, p > 0.05).

Marginal analysis was performed, holding cognitive baselines at the average normalized cognitive baseline for each disease (PD = 0.71, AD = 0.64) and predicting outcomes across the range of subiculum connectivity values. Permutation test was then used to assess if the difference between outcomes across the levels of cognitive baseline. There was no significant difference in GLM predictions across the levels of cognitive baseline (PD: Δβ_Baseline_ = 0.01, p > 0.05; AD: Δβ_Baseline_ = -0.07, p > 0.05).

1.5.5 Specificity of Interaction Between Subiculum and Age on Cognition

Within PD patients, we recomputed the general linear model, replacing age with a different clinical covariate and recomputed the spatial correlation each time. Age was replaced baseline MDRS, baseline UPDRS-3, and patient identification number, but none demonstrated significant spatial correlation to the AD response topology (p > 0.05). To assess neuroanatomical specificity to the memory ROI, we replaced the memory ROI with connectivity to several other ROIs (cerebellar vermis, occipital lobe, putamen, pons, frontal lobe, and motor cortex), and found none demonstrated significant spatial correlation to the AD joint distribution (p > 0.05). Finally, to assess specificity to cognition, we replaced MDRS with UPDRS-I and found no significant spatial correlation to the AD joint distribution (p > 0.05).

1.5.6 Results Reproduce at Whole-Brain Level

We next evaluated if the observed correlation differences in age-based subgroups were statistically robust. We binned patients into 10 subgroups ranging from 60-70, finding the age groups had overall significantly different correlations with subiculum connectivity across all 10 bins (min ΔR = -0.47, min p = 0.045). These results were also robust to using Spearman’s Rho (min ΔRho = -0.52, min p = 0.04).

Given our primary analyses focused on connectivity between VTAs and the subiculum, we investigated if our results reproduced within whole-brain networks. We therefore repeated our analyses, examining connectivity between VTAs and all brain voxels. The whole-brain networks of connections covarying with cognitive outcomes in PD and AD (Supplementary Figure 7) were more similar than expected by chance (p < 0.05). Additionally, the whole-brain connections interacting with age across PD and AD involved several FWE-significant regions (Supplementary Figure 8A), with the peak being in the subiculum (Supplementary Figure 8B).

To further assess the neuroanatomical specificity of the interaction, we performed a voxelwise general linear model regressing age, voxel connectivity to the subiculum, and their interaction upon cognitive improvement. We then extracted the voxelwise T-value of the interaction coefficient, generating an interaction map. We generated an interaction map for PD and AD, and then measured their spatial correlation to the subiculum connectivity profile (Supplementary Figure 9). To assess significance of the spatial correlation to the memory network, permutation of the patients and recomputation of the interaction map was conducted 10 000 times. This derived the probability that the similarity occurred by chance. Both the PD and AD interaction maps were more similar to the memory network than expected by chance (PD: Spatial Correlation = 0.49, p < 0.001; AD: Spatial Correlation = 0.25, p < 0.05.

Finally, we then performed a voxelwise mixed effects model to identify regions that interact across both diseases after accounting for the variance in each disease’s slope and intercepts. Voxelwise significance was derived by Bonferroni correcting the voxelwise p-values to achieve family-wise error correction. All regions of FWE-significant shared interaction fell within or overlapped with the memory network (Supplementary Figure 10).

Supplementary Figure 7. Brain networks associated with cognitive outcomes in PD (top) and AD (bottom) have similar topology but opposite sign. Each map represents the voxelwise correlation between cognitive outcome and VTA connectivity. Each patient’s VTA was seeded to derive that VTA’s whole-brain connectivity. The spatial correlation of the maps was more similar than expected by chance, but with opposite signs (p < 0.01). The coloring of the Parkinson Cognitive Correlation Network is flipped to facilitate comparison to the AD map.

Supplementary Figure 8. Whole-brain voxel-wise map showing the interaction between age, VTA-connectivity, and cognitive outcomes across both discovery PD and AD DBS datasets. A) The unthresholded T-map of interactions between age, connectivity, and cognitive outcomes. Significant interactions (p_FWE_ < 0.05) spanned the cerebellum, hippocampus, subiculum, orbitofrontal cortex, prefrontal cortex, caudate, and precuneus. This analysis is the same as the analysis in Figure 4 (interaction of subiculum connectivity, age, and cognitive outcomes), but carried out across the whole brain. B) The whole-brain peak of the interaction between age, connections, and cognitive outcomes with the subiculum region of interest overlaid. Results are unchanged including the PD-validation cohort.


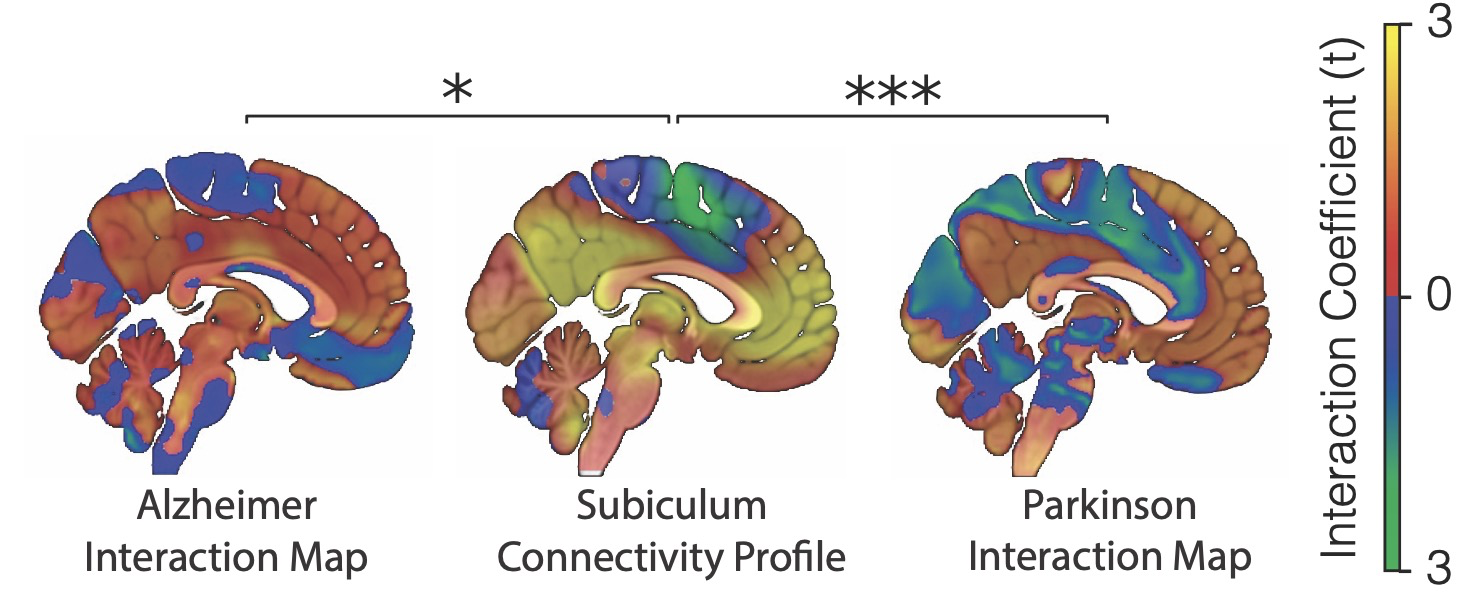


Supplementary Figure 9. PD and AD Interaction Maps Share Topology with Subiculum Connectivity Profile. Both the PD and AD interaction maps were more similar to the memory network than expected by chance (PD: Spatial Correlation = 0.49, p < 0.001; AD: Spatial Correlation = 0.25, p < 0.05).


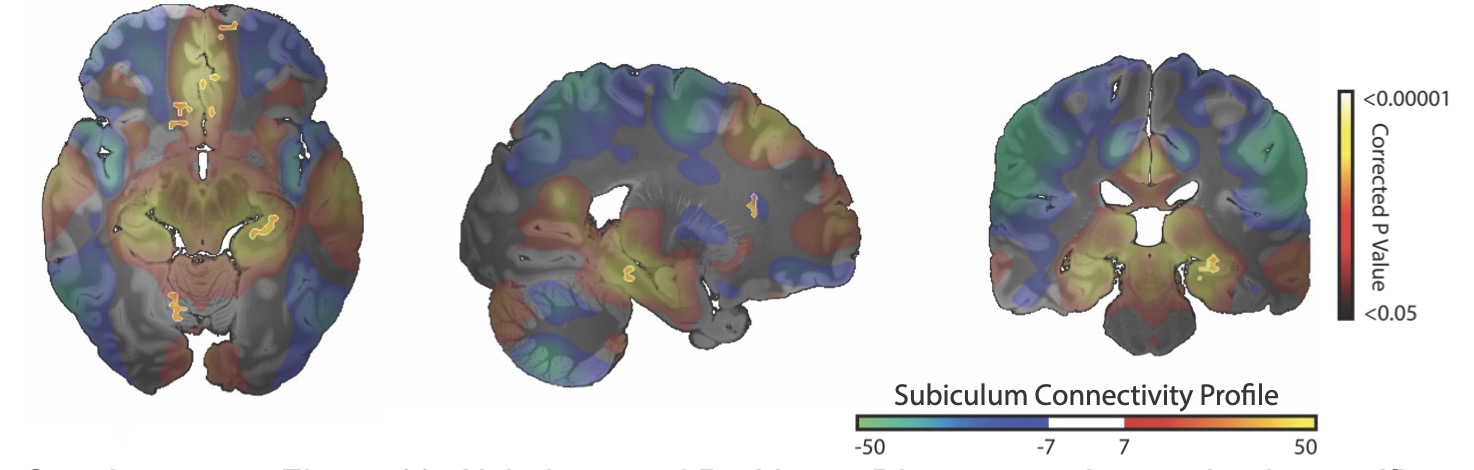


Supplementary Figure 10. Alzheimer and Parkinson Disease age interaction is specific to the memory network. The whole-brain connections of the subiculum (memory network) is shown in green/yellow, thresholded to regions of statistical significance (absolute t-values >7). A whole-brain mass univariate mixed effects model was used to find connections interacting with age after accounting for mixed effects. Connections where both cohorts similarly interact with age are shown outlined in white. The interacting connections occur exclusively within the memory network.

1.6 Age Inflection Point is Not Driven by Differences in Groups

Inflection point in age and subiculum connectivity were algebraically solved by setting Supplementary Equations 3 to zero and solving for age. For visualization of inflection points in age, we calculated the partial gradients of the response surface (Supplementary Figure 11).

We next evaluated the distributions and central tendencies of age to see if shared traits may be driving the similar inflection points (Supplementary Figure 12). Median age differed significantly (Mann-Whitney U-Test, U = 93, p < 0.0001). Distribution of age differed significantly across cohorts (Kolmogorov-Smirnov = 0.25, p < 0.05).

Next, we split patients into subgroups by their age inflection points. We checked if differences in connectivity were introduced by the subgrouping process (Supplementary Figure 13). We found no difference in the PD subgroups (p > 0.05), nor in the AD subgroups (p > 0.05).

Supplementary Figure 11. Visualization of the inflection of age of using partial gradients. A) The partial derivative of age across all values of connectivity for PD. B) The partial derivative of age across all values of connectivity for AD. The white band where the partial gradient reaches zero represents the inflection point.


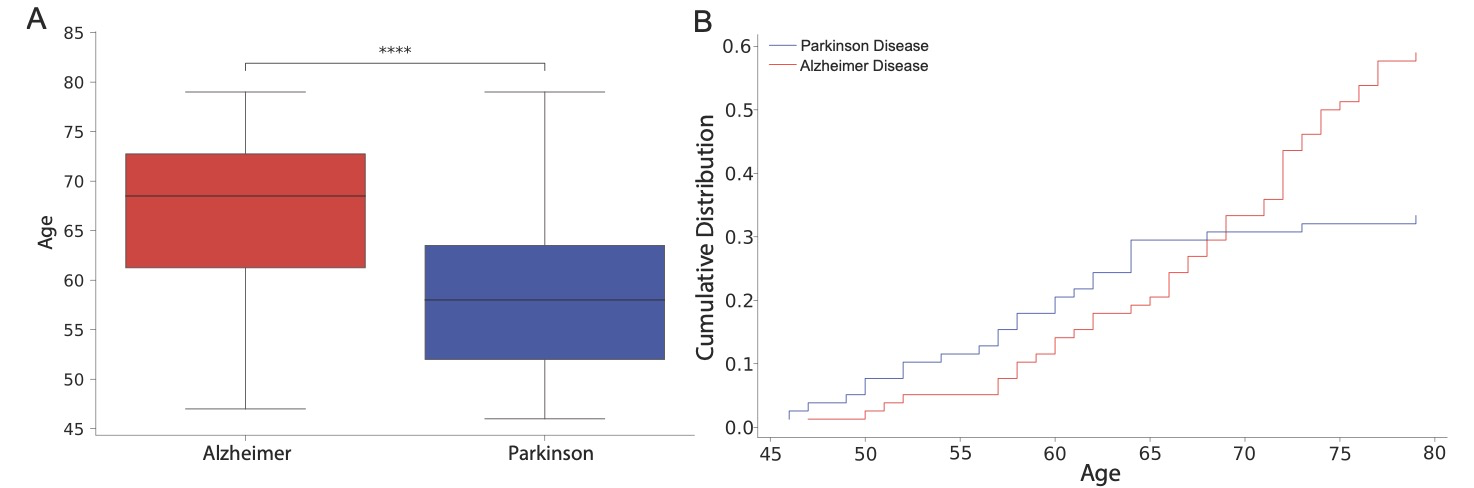


Supplementary Figure 12. Cohort distributions and central tendencies do not drive the inflection point similarity. A) Comparison of median age. Median age differed significantly across groups (U = 93, p < 0.0001) B) Cumulative distribution function of age. Distribution of age differed significantly across cohorts (Kolmogorov-Smirnov = 0.25, p < 0.05).


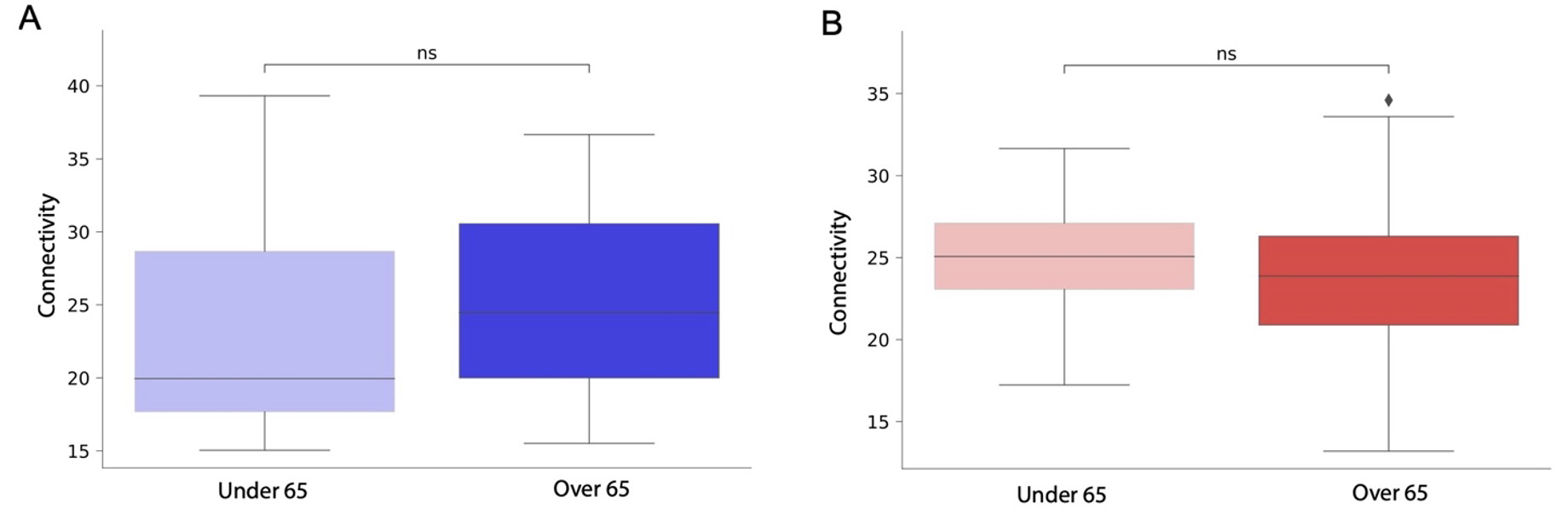


Supplementary Figure 13. Connectivity does not differ across age inflection subgroups. A) Comparison of PD mean subiculum connectivity across age groups. There was no significant difference between means (T-stat = 0.06, p > 0.05). B) Comparison of AD mean subiculum connectivity across age groups. There was no significant difference between means (T-stat = 0.03, p > 0.05).

1.7.1 Identification of Inflection Points in Connectivity

The inflection points in connectivity can be directly solved for using Supplementary Equations 1-3. These can be applied to the equation identified by the GLM relating connectivity, age, and their interaction to cognitive outcomes. This must be done separately for each cohort, giving the connectivity inflection for each cohort. Technically, when deriving the inflection in age, the inflection in connectivity must simultaneously be solved for. However, for simplicity, we first deriving the inflection in age and demonstrating how it can be used to simplify our data. Having established that foundation, we now turn to the connectivity inflection point.

While the inflection can be derived algebraically, it is often useful to visualize it directly. By modelling the response surface (the predicted outcomes from the general linear model), we can take the derivative of that response surface. When we take the partial derivatives, only deriving the connectivity axis or age axis, we get the partial gradients of the response topology.

1.7.2 Connectivity Inflection Point Similarity is not Driven by Cohort Similarities

The connectivity inflection points in PD (T = 24.2) and AD (T = 23.8) were very similar. It was possible that this was simply driven by the underlying connectivities sharing a similar distribution, and there for sharing an inflection point. Or, perhaps this was driven by their central tendencies being very close. To address this, we performed central tendency testing and distribution testing (Supplementary Figure 14). However, we found the central tendencies (medians) of the PD and AD discovery cohorts were significantly different (p < 0.05), as were their distributions (p < 0.05).

We also ensured there was no artifactual difference in age that was introduced by splitting groups by connectivity (Supplementary Figure 15). After splitting PD and AD into connectivity-based subgroups, we measured the mean age value across the low and high connectivity subgroups, finding no difference between the PD subgroups (p > 0.05), nor AD subgroups (p > 0.05).

**

Supplementary Figure 14. Cohort central tendencies and distributions do not drive the connectivity inflection similarity. A) Comparison of median connectivity. Median connectivity differed significantly across groups (U = 95, p < 0.05) B) Cumulative distribution function of connectivity. Distribution of age differed significantly across cohorts (Kolmogorov-Smirnov = 0.23, p < 0.05).

**
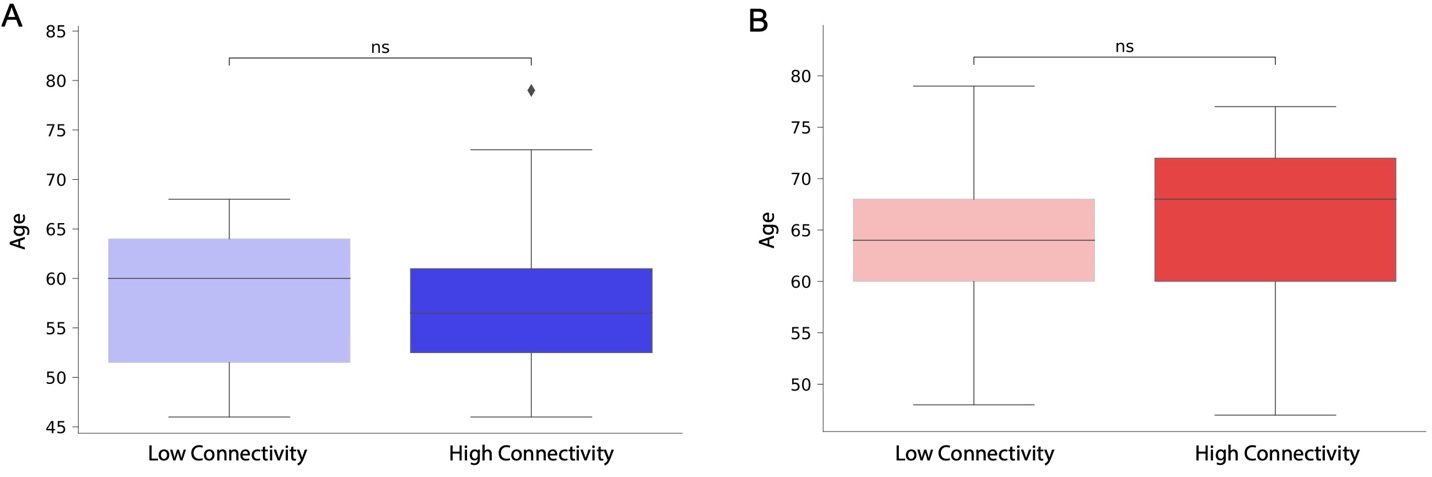
**

Supplementary Figure 15. Age does not differ across subgroups defined by connectivity inflection point. A) Comparison of PD mean age across connectivity groups. There was no significant difference between means (T-stat = 0.07, p > 0.05). B) Comparison of AD mean age across connectivity groups. There was no significant difference between means (T-stat = 0.32, p > 0.05).

1.7.3 Connectivity Can Also Resolve the Paradox

We next recreated our initial delta-R analysis that used age. First, we performed our analysis without splitting by the connectivity inflection point (Supplementary Figure 16, top). There was no significant difference between the PD groups (p > 0.05), PD discovery and AD discovery (p > 0.05) nor AD discovery and PD validation (p > 0.05). This acts as a control analysis demonstrating the delta-R analysis will not find spurious differences between groups.

Next, we binned the patients by the connectivity inflection point performed the delta-R analysis within the high connectivity subgroups across cohorts (Supplementary Figure 16, middle). Age was positively correlated to cognitive outcomes in all groups, with no significant difference across the diseases (p > 0.05). We repeated this analysis in the low connectivity group, finding that all cohorts had better cognitive outcomes as age decreased, with no difference across diseases (p > 0.05).

Having established that there was no difference in how patients responded *within* connectivity groups, we next wondered if there was a difference *across* connectivity groups (Supplementary Figure 16, bottom). Overall, a delta-R analysis contrasting the high connectivity group and low connectivity group found a significant difference (p < 0.05). We next evaluated the difference in each cohort’s responses. There was a difference between PD discovery (p < 0.05), AD discovery (p < 0.05), and the PD validation (p < 0.05).

Next, we categorized patients into connectivity inflection point subgroups. We performed an ANCOVA, controlling for the effect of age and isolating the effect of the connectivity subgroups upon cognitive outcomes. We found a significant interaction between connectivity group and age (Supplementary Figure 17A, C, p < 0.05). We then plotted the average outcomes of age-optimized patients (e.g. young and low connectivity) to non-optimized patients (e.g. young and high connectivity, Supplementary Figure 17B). Using planned contrasts of the ANCOVA, we found young low-connectivity patients outperformed their high-connectivity counterparts (p < 0.05), as did the older patients with high connectivity compared to their low connectivity counterparts (p < 0.05). To visualize this, we created an estimated marginal means plot, which allows us to project the expected outcomes, ‘adjusted’ by the ANCOVA. For this, we projected the outcomes of patients in old age, but then modeled outcomes across low and high connectivity situations for each cohort. We again contrasted these connectivity groups, finding a significant difference between how low and high connectivity patients were expected to perform old age (p < 0.05).

Further, these same results were reproduceable when using a mixed-effects model which more appropriately accounts for the variance inherent to the different cohorts (p < 0.05). This interaction was stable whether using mixed effects, fixed effects, controlling for cohort as a covariate, or not controlling for cohort as a covariate.


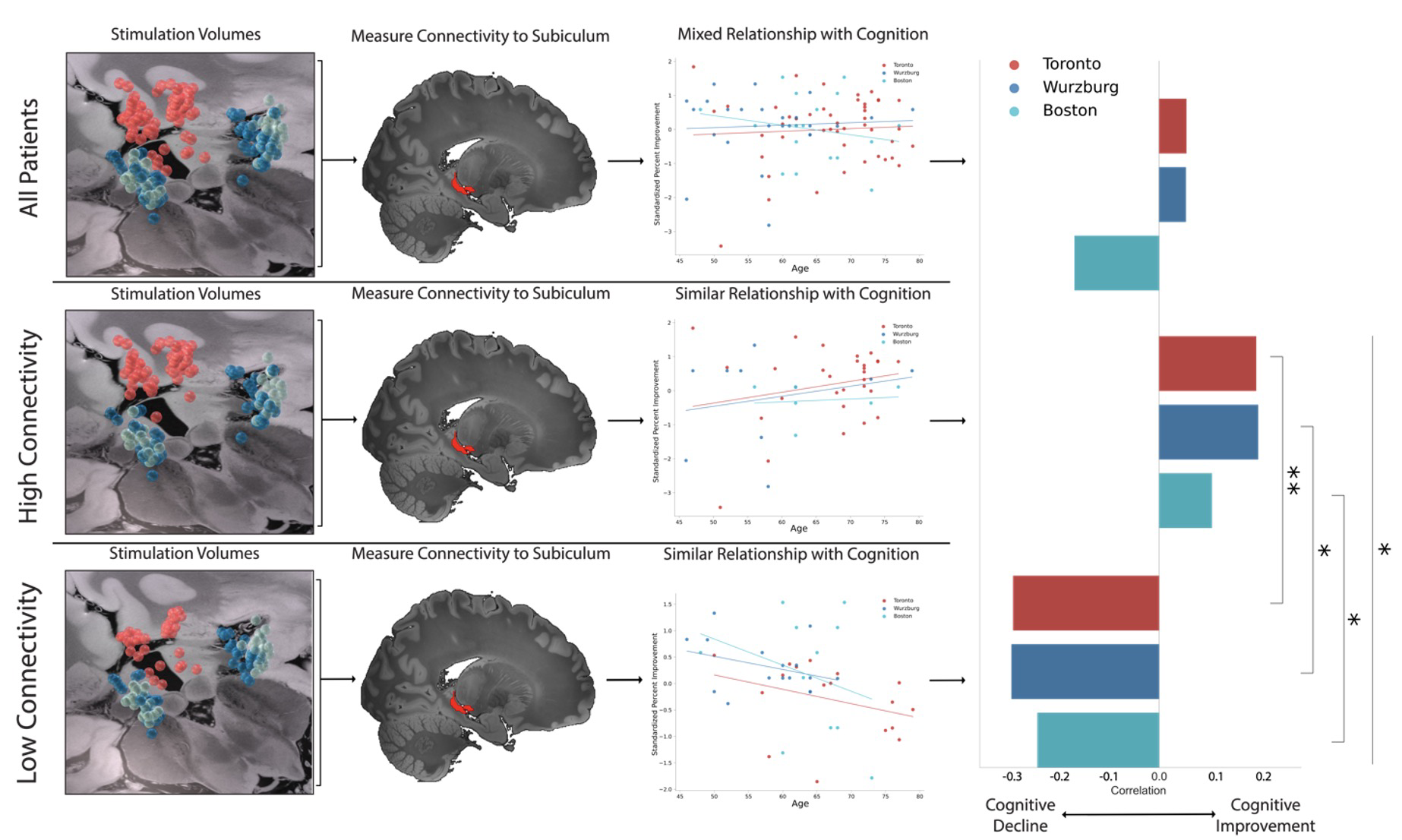


Supplementary Figure 16. Connectivity Groups Across Diseases Respond Differently to Subiculum Connectivity. Top) All patients. Age does not significantly correlate with cognitive outcomes in all-comers, nor is it significantly different across groups (p > 0.05). Middle) Patients highly connected to the subiculum. Age positively correlates with cognitive outcomes in all cohorts. Bottom) Patients minimally connected to the subiculum. Age negatively correlates with cognitive outcomes in all cohorts. There is a significant difference in how high and low connectivity groups respond as per an overall contrast (p < 0.05). There is a significant difference between high and low groups in the AD discovery cohort (p < 0.01), the PD discovery cohort (p < 0.05), and the PD validation cohort (p < 0.05). There was no significant difference between cohorts within connectivity groups (p > 0.05).


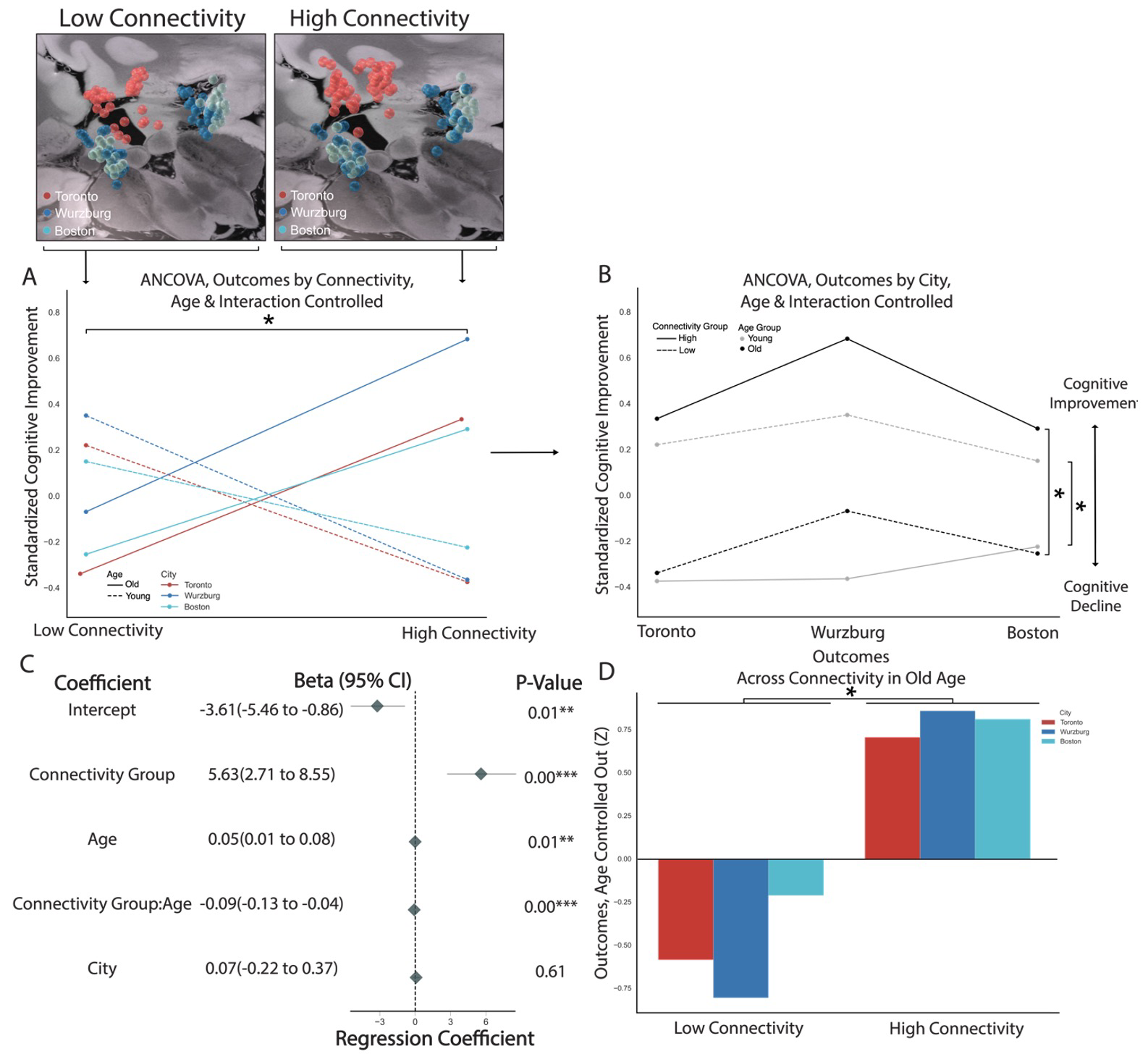


Supplementary Figure 17. ANCOVA-based analysis finds significant differences in average cognitive outcomes across the connectivity subgroups. A) Interaction plot demonstrating average patient outcomes across each subgroup. A significant interaction between connectivity group and age was detected by ANCOVA. B) Main effects plot, demonstrating the same average outcomes, but visualized across cohorts. The planned contrasts (derived from the ANCOVA) *within* connectivity groups *across* age levels are highlighted with significance bars. C) Forest plot representing the results of the ANCOVA. D) Estimated marginal means plot, projecting the situation wherein low and high connectivity patients from each cohort are of an older age. This allows for ‘adjusting’ the effect of age out, isolating how these patients are expected to respond just due to their connectivity subgroup. Contrast of these outcomes demonstrated a significant difference between connectivity groups (p < 0.05). In A and B, patients are also split into age subgroups; this is based on the inflection point in age, and is done simply for eased visualization of the ANCOVA.

1.8.1 Sensitivity Analyses for Age & Connectivity Split

Having added in the validation cohort to our analyses, we first verified that our original findings held. We repeated our initial analysis finding an interaction between age and connectivity, *without subgrouping by inflection points*. We performed our fixed effects analysis using general linear modelling, regressing age, connectivity, and their interaction upon cognitive outcomes. We found a significant interaction (p < 0.05) when considering all patients in a mega-analysis (not controlling for cohort as a covariate), and when controlling for cohort as a covariate (p < 0.05). This interaction was still significant when using a mixed effects model which more appropriately controlled for the random variance in mean and random variance in how the groups might respond to the regressors (p < 0.05).

Having demonstrated our original interactions were stable and consistent with the new validation cohort, we applied both inflection points. Thus, all cohorts were split by age and connectivity inflection points, resulting in four subgroups. However, to avoid circularity, we used the inflection points form the AD discovery cohort to subgroup the PD cohorts, and the PD discovery cohorts to subgroup the AD cohort.

For visualization, we apply our 2-way ANOVA, plotting both the average cognitive outcomes within each cohort as well as the ‘adjusted’ estimated outcomes from the ANOVA. This 2-way ANOVA demonstrates the adjusted outcomes expected by the model, demonstrating a significant interaction (Supplementary Figure 18A), the contrast between connectivity levels within an age group (Supplementary Figure 18B), and the results of the model (Supplementary Figure 18C). Further, it also demonstrated the unadjusted (actual) cognitive outcomes of the patients in these subgroups (Supplementary Figure 18D), their contrast (Supplementary Figure 18E). This interaction between age and connectivity groups was significant whether controlling for cohorts or not, as well as in a 3-way ANOVA accounting for potential cohort interactions, and in a mixed-effects analysis (p < 0.05).

We also abandoned statistical models and simply evaluated this interaction across all patients using t-tests (Supplementary Figure 18F). For younger patients, cognitive outcomes were found to be significantly higher in the group with low connectivity (Z-scored outcomes: 0.28±0.09) versus the high connectivity (Z-scored outcomes: -0.37±0.32) group (p = 0.046). For older patients, cognitive outcomes were found to be significantly higher in the high connectivity (Z-scored outcomes: 0.38±0.14) compared to the low connectivity (Z-scored outcomes: -0.32±0.17) group (p = 0.0044). Further, young patients with low connectivity also had higher outcomes than older patients with low connectivity (p < 0.05). Older patients with high connectivity also outperformed young patients with high connectivity (p < 0.05).


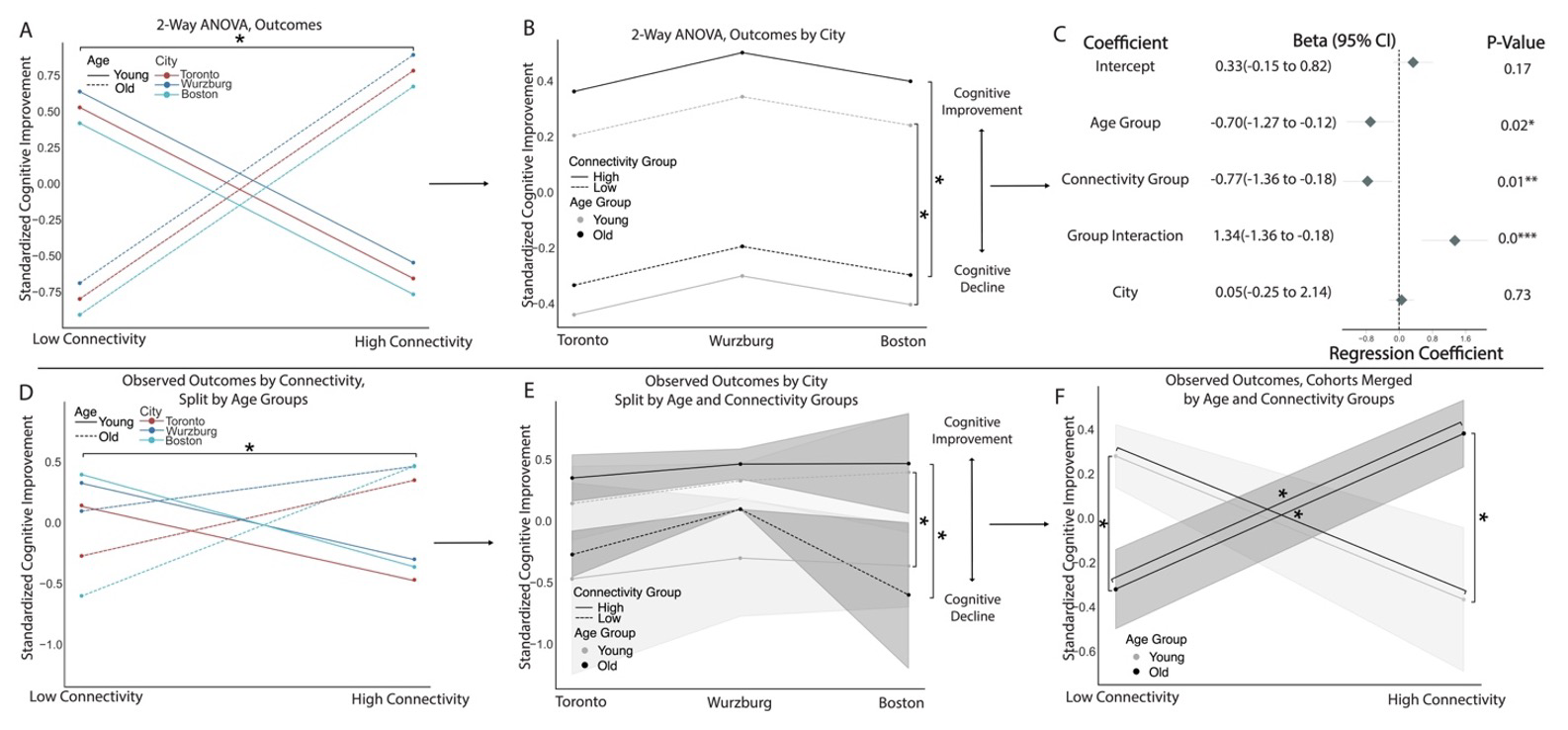


Supplementary Figure 18. Age and connectivity subgrouped patients demonstrate findings robust to multiple analyses. A) Interaction plot of the average cognitive outcomes across the subgroups of each cohort, adjusted using estimated outcomes from the 2-way ANOVA. This demonstrates the significant interaction between age and connectivity subgroups. B) A main effects plot, demonstrating the same estimates from the 2-way ANOVA for each cohort. This highlights the planned contrast between old and young patients across their connectivity levels, which demonstrated older patients across all cohorts perform best if they have high connectivity (p < 0.05) while the opposite is true for young patients (p < 0.05). C) The 2-way ANOVA represented as a forest plot. D) An interaction plot of the actual average patients outcomes, split by subgroups. This demonstrates again the significant interaction between groups (p < 0.05). E) A main effects plot of actual average cognitive outcomes across each subgroup, demonstrating the contrast which was found to be positive in the planned contrast. F) A mega-analysis, where all cohorts were combined. All older patients with high connectivity perform better than older patients with low connectivity (2-sample t-test, p < 0.05), and all younger patients performed best with low connectivity (2-sample t-test, p < 0.05). Further, older patients with high connectivity perform better than young patients with high connectivity (2-sample t-test, p < 0.05) and vice-versa (2-sample t-test, p < 0.05). Multiple comparisons were corrected with Tukey’s Honestly Significant Difference.

1.9.1 Overall Explanatory Power

The mediation analysis was significant across all cohort (p = 0.0317), and was robust to fixed effects, mixed effects, and controlling for cohort. It was also significant within the PD discovery cohort (p = 0.0212), the AD cohort (p = 0.012), and the PD-discovery cohort (p = 0.039).

We next evaluated the overall explanatory power of the models with a series of hierarchical regressions. We tested if any individual variable was providing significant explained variance in cognitive outcomes, or if it was due to an interaction of variables (Supplementary Table 2). Among age, subiculum connectivity, and hippocampal volume, found no individual variable explained substantial variance (p_min_ = 0.29). Age and connectivity explained significant variance across all patients (p = 0.023).

As well, hippocampal volume and connectivity explained significant variance (R^2^ = 0.14, p = 0.011), which increased when age was included (R^2^ = 0.33, p = 0.032). This was specific to age, hippocampal volume, and subiculum connectivity, and similar explained variance was not possible with other demographic variables.

We tested if this effect is specific to age, connectivity, and hippocampal volume. Replacing any of these variables with other demographic variables considerably reduced explained variance despite similar number of regressors. Upon replacement of the original variables, the regressions were no longer significant.

Supplementary Table 2. Hierarchical regressions demonstrate age, connectivity, and hippocampal volume are all required to explain variance in cognitive outcomes. Significant regressions are bolded.

| **Regression** | **Under 65 (R^2^)** | **Over 65 (R^2^)** | **All Comers (R^2^)** |
| --- | --- | --- | --- |
| Age | 0.01 | 0.09 | 0.00 |
| Connectivity | 0.02 | 0.04 | 0.02 |
| Hippocampal Volume | 0.00 | 0.02 | 0.00 |
| Age & HPC Volume | 0.09 | 0.09 | 0.04 |
| Age & Connectivity | 0.10 | 0.12 | **0.14** |
| HPC Volume & Connectivity | **0.18** | **0.23** | **0.16** |
| Age & Connectivity & HPC Volume | **0.27** | **0.38** | **0.33** |
